# Supplementary material for: Analysis and correction of compositional bias in sparse sequencing count data
Source: BMC Genomics. 2018 Nov 6;19:799. doi: 10.1186/s12864-018-5160-5 (PMC6219007; doi:10.1186/s12864-018-5160-5)
Supplement: Supplementary file 1 — Supplementary Note. Presents further discussions on compositional bias, and supplementary results in context. (PDF 17,810 kb) [file 12864_2018_5160_MOESM1_ESM.pdf]

Supplementary Information

Analysis and correction of compositional bias in sparse sequencing count data

M. Senthil Kumar[*,1,2], Eric V. Slud[2,3], Kwame Okrah[4], Stephanie Hicks[5,6], Sridhar Hannenhalli[2], Héctor Corrada Bravo[2]

[1]Graduate program in Bioinformatics, University of Maryland, College Park, MD 20740.

[2]Center for Bioinformatics and Computational Biology, University of Maryland, College Park, MD 20740.

[2]Department of Mathematics, University of Maryland, College park, MD 20742.

[3]Center for Statistical Research and Methodology, U.S Census Bureau, Suitland, MD 20746.

[4]GRED Oncology Biostatistics, Genentech, San Francisco, CA 94080.

[4]Biostatistics and Computational Biology, Dana-Farber Cancer Institute, and

[6]Biostatistics, Harvard T.H. Chan School of Public Health, Harvard University, Boston, MA 02215

[*]Email: MSK: smuthiah@umiacs.umd.edu

October 1, 2018

1 Without compositional correction, unbiased DE inference is possible only under narrow conditions: a bad sign for RPKM/Rarefication based normalization approaches.

We now ask how inference of differential expression is confounded when sequencing data is directly subjected to a generalized linear model (a framework used by most genomic data analysis packages) with the most natural normalization involving total number of sequencing reads generated per sample.

Notation: We use i, j and g to index features, samples and experimental conditions/groups respectively. We choose $g = 1$ as our control group. A $\cdot$ in a subscript represents vectorized quantities: for instance if Z_{gji} represents an object corresponding to the ith feature, j^{th} sample and the gth group, $Z_{g\cdot i}$ represents the vector $[|Z_{gji}|]_{j=1}^{N}$ for all samples $j = 1,\ldots N$. Similarly, $Z_{gj\cdot}$ would correspond to the vector $[|Z_{gji}|]_{i=1}^{p_{tot}}$ across all features $i = 1\ldots p_{tot}$. We will also use the bar notation: $\overline{Z_{g+i}}$ to represent sample-wise average of entry i in group g.

We consider samples $j = 1\ldots n_g$ from groups $g = 1\ldots G$. We let A_g denote the set of features truly expressed in the samples from group g (regardless of them being observed or not), and use $U_g = A_g - A_1$ as the set of features expressed in group g but not in the control group. We only consider features in the set A_1, and index them with $i = 1\ldots p = |A_1|$. For interestingness, we assume $p > 1$.

We imagine the the following process:

$$X_{gj\cdot}^{0} \overset{\text{technical variation}}{\longrightarrow} X_{gj\cdot} \overset{\text{sequencing}}{\longrightarrow} Y_{gj\cdot}, \tag{1}$$

where $X_{gj\cdot}^{0}$ and $X_{gj\cdot}$ is a p_{tot} length vector with each ith slot containing the absolute abundances before and after technical perturbations respectively. With $1_{p_{tot}}$ representing a p_{tot} length vector of 1s, let $T_{gj}^{0} = 1_p^T X_{gj\cdot}^{0}$ and $T_{gj} =$

$1_p^T X_{gj\cdot}$ denote the respective total abundances. We shall assume, $X^0_{gj\cdot}|T^0_{gj}, q^0_{g\cdot} \sim Multinomial(T^0_{gj}, q^0_{g\cdot})$, and $X_{gj\cdot}|T_{gj}, q_{g\cdot} \sim Multinomial(T_{gj}, q_{g\cdot})$, where $q^0_{g\cdot}$ and $q_{g\cdot}$ is the p_{tot}-length relative abundance vector of features that all samples within a group share. Similarly, at sequencing depth $\tau_{gj} = Y_{gj+}$, we assume $Y_{gj\cdot}|\tau_{gj}, X_{gj\cdot} \sim Multinomial(\tau_{gj}, X_{g\cdot}/X_{gj+})$ are the sequencing counts obtained by sequencing $X_{gj\cdot}$. This has a marginal expectation $E[Y_{gji}|\tau_{gj}] = q_{gi}\tau_{gj}$, so averaging over the observed proportions $\hat{q}_{gji} = Y_{gji}/\tau_{gj}$ has an expectation $E[\overline{\hat{q}_{g+i}}] = q_{gi}$. Clearly, every i^{th} slot of $q_{1\cdot}$, the vector of control group feature proportions is such that $0 < q_{1i} < 1$. Fold changes are ratios of marginal expectations: we use $\nu^0_{gi} = E[\overline{X_{g+i}}^0]/E[\overline{X_{1+i}}^0]$, $\nu_{gi} = E[\overline{X_{g+i}}]/E[\overline{X_{1+i}}]$. Denoting $E[T_{g1}]$ as the marginal average total abundance of the total abundances T_{gj}, we have $E[X_{gji}] = E[T_{g1}]q_{gi}$ for all $j = 1 \ldots n_g$. Describing $E[T^0_{g1}]$ similarly, the aforementioned fold changes can be re-written as: $\nu^0_{gi} = E[X^0_{g1i}]/E[X^0_{11i}]$, $\nu_{gi} = E[X_{g1i}]/E[X_{11i}]$. Similarly, we let $\xi_{gi} = q_{gi}/q_{1i}$ represent the fold changes of feature i of relative abundances (i.e., Y adjusted for sequencing depth) respectively. Furthermore, we set $\phi_g = \sum_{i \in U_g} q_{gi}$, the summed proportion of features internally expressed only in group g relative to the control group (regardless of whether they are observed or not). In the entire process, we only get to observe $Y_{gj\cdot}$ for all $j = 1 \ldots n$ and $g = 1 \ldots G$.

The above are our modeling assumptions.

Lemma 1.1. *Under the model above, for all features $i = 1 \ldots p$, the fold changes computed from relative abundances at stage Y, ξ_{gi}, equal those of absolute abundances at stage X, ν_{gi}, if and only if $\frac{E[T_{g1}]}{E[T_{11}]} = 1$.*

Proof. The proof follows directly from the definition of fold changes ν_{gi} associated with the ith feature's absolute abundances.

$$\nu_{gi} = \frac{E[X_{g1i}]}{E[X_{11i}]} = \frac{E[T_{g1}]q_{gi}}{E[T_{11}]q_{1i}} \equiv \Lambda_g \cdot \frac{q_{gi}}{q_{i1}} = \Lambda_g \xi_{gi} \tag{2}$$

which is equal to ν_{gi} iff $\Lambda_g = 1$. □

Lemma 1.2. *Let $\Theta_g = \{q_{1\cdot}, \nu_{g\cdot}, \phi_g, \Lambda_g\}$, where $\Lambda_g = E[T_{g1}]/E[T_{11}]$. Under the above generative process, and the standard log-linear mean model on the total sum normalized data $\log E[Y_{gji}/\tau_{gj}] = \mu_i + \alpha_{gi}$ with μ_i estimating logged control group proportions, $\log q_{1i}$, and α_{gi} estimating the log-fold change of relative abundances, $\log \xi_{gi}$, there exists a unique constraint on Θ_g, under which $\alpha_{gi} = 0 \iff \log \nu_{gi} = 0$, the log- fold change associated with absolute abundances. Furthermore, this constraint is given as:*

$$\frac{1}{1 - q_{1i}} \left[\Lambda_g \phi_g + \sum_{k, k \neq i} \nu_{gk} q_{1k} \right] = 1$$

Proof. Following Lemma 1.1, re-write the proportion in group g as:

$$q_{gi} = \Lambda_g^{-1} \nu_{gi} q_{1i} = \frac{\Lambda_g^{-1} \nu_{gi} q_{1i}}{1} = \frac{\Lambda_g^{-1} \nu_{gi} q_{1i}}{\phi_g + \sum_{k \in A_1} q_{gk}} = \frac{\nu_{gi} q_{1i}}{\Lambda_g \phi_g + \nu_{gi} q_{1i} + \sum_{k \in A_1, k \neq i} \nu_{gk} q_{1k}} \equiv \frac{1}{1 + \frac{\nu_{g \setminus i}}{\nu_{gi}} \frac{(1 - q_{1i})}{q_{1i}}} \tag{3}$$

and

$$\nu_{g \setminus i} = \frac{1}{1 - q_{1i}} \left[\Lambda_g \phi_g + \sum_{k, k \neq i} \nu_{gk} q_{1k} \right] \tag{4}$$

Substituting eqn. 3 in the assumed model: $\log E[Y_{gji}|\tau_{gj}] = \log q_{gji} + \log \tau_{gj} = \mu_i + \alpha_{gi} + \log \tau_{gj}$, and noting $\mu_i = \log q_{1i}$, $\alpha_{gi} = \log \frac{q_{gi}}{q_{1i}}$, it is clear that $\alpha_{gi} = 0 \iff \frac{\nu_{g \setminus i}}{\nu_{gi}} = 1$. Thus, only when $\nu_{g \setminus i} = 1$, $\alpha_{gi} = 0 \iff \nu_{gi} = 1$. □

Theorem 1.3. *Under the above model, there exists a unique vector of fold changes $\nu^*_{g\cdot}$ under which $\forall i = 1 \dots p$, $\alpha_{gi} = 0 \iff \nu_{gi} = 1$. Furthermore, each $i = 1 \dots p$ entry of $\nu^*_{g\cdot}$ is given as:*

$$\nu^*_{gi}(\Lambda_g, \phi_g, q_{1\cdot}) = \left[\left(\frac{1-q_{1i}}{q_{1i}} \right) \left(\frac{\eta_g}{1-q_{1i}} - 1 \right) + \left(\frac{1}{1-p} \right) \sum_{k \in A_1} \left(\frac{1-q_{1k}}{q_{1k}} \right) \left(\frac{\eta_g}{1-q_{1i}} - 1 \right) \right] \tag{5}$$

with $\eta_g = \Lambda_g \cdot \phi_g$.

Proof. We want to study the conditions under which $\nu_{gi} = 1 \forall i \in A_1$. Substituting this in equation 4 from lemma 1.2, and stacking the constraints for all i, we get a linear system:

$$Q\nu = \gamma$$

where, Q is a $p \times p$ matrix with $Q(i,j) = \frac{q_{1j}}{1-q_{1i}}$ if $j \neq i$ and 0 otherwise. $\nu = [|\nu_{gi}|]_{i=1}^p$, a $p \times 1$ vector, and $\gamma = [|\gamma_{gi}|]_{i=1}^p$, a $p \times 1$ column vector with $\gamma_{gi} = 1 - \frac{\eta_g}{1-q_{1i}}$, where $\eta_g = \Lambda_g \phi_g$, a non-dimensional parameter. A solution for this equation is obtained directly as $\nu^* = [|\nu^*_{gi}|]_{i=1}^p = Q^{-1}\gamma$ if Q is invertible. Notice that $Q = rq_{1\cdot}^T - D$ where r is a $p \times 1$ vector with the ith component equal to $\frac{1}{1-q_{1i}}$ and $q_{1\cdot}$ is a $p \times 1$ vector of control proportions. D is a $p \times p$ diagonal matrix with diagonal entries given by $\frac{1-q_{1j}}{q_{1j}} \forall j = 1 \dots p$. If we set $F = D - rq_{1\cdot}^T$, we then want $Q^{-1} = -F^{-1}$. Obtaining F^{-1} is easy. Denoting $U = -r$, and $V = q_{1\cdot}^T$, we can write, $F^{-1} = (D + UV)^{-1}$. Woodbury identity then yields $F^{-1} = D^{-1} - D^{-1}U(I + VD^{-1}U)^{-1}VD^{-1}$, a $p \times p$ matrix with $F^{-1}(i,j) = \frac{1-q_{1j}}{q_{1i}} \left(\frac{1}{1-p} \right)$ if $i \neq j$, and $\frac{1-q_{1i}}{q_{1i}} \left(1 + \frac{1}{1-p} \right)$ if $i = j$. The exact solution for the fold changes satisfying the linear constrains are then given by $\nu^*_{g\cdot} = -F^{-1}\gamma$, with ν^*_{gi} given by eqn. 5 above. □

Theorem 1.4 (Validity of Total Sum Normalization in Reconstructing X)**.** *Under the modeling assumptions above, the vector of feature-wise fold changes under which total sum normalization can yield unbiased inferences (correct fold changes and non-zero significance) of absolute abundances of all $i = 1 \dots p$ features in group g at stage X is given by $\nu^*_g(1, \phi_g, q_1)$, where $\nu^*_g(\Lambda_g, \phi_g, q_1)$ is defined in Theorem 1.3.*

Proof. Proof follows directly from Lemma 1.1 and Theorem 1.3. □

The last result was also verified numerically. As an example, suppose $q_{1\cdot} = [0.25, 0.25, 0.1, 0.1, 0.3]^T$. For $\Lambda_g = 1$, and $\phi_g = 0.05$, the fold changes that need to be achieved for unbiased inference is given by: $\nu^*_{g\cdot} = [0.95, 0.95, 0.88, 0.88, 0.96]^T$ implying that downregulation across features can be detected well as the unique features will compete for sequencing output. For $\Lambda_g = 1$, and $\phi_g = 0.4$, no feasible solution exists. (In such situations, an approximate solution can be obtained by solving the convex problem $\arg\min_{\nu:\nu \geq 0} ||Q\nu - \gamma||_2$. Because Q is invertible, a least squares solution is obtained and any component $\nu_{gi} < 0$ in the solution vector is replaced with a value of 0. But we do not explore these ideas any further in this work). For the case $\phi_g = 0$, the optimal solution is trivial: $\nu_{gi} = 1$ for all i i.e., no perturbation in any of the features. Providing additional constraints by fixing at least one of the fold changes yields the single, constrained solution on the rest of the fold changes: the solution vector ν^* is obtained by replacing $\eta_g = \Lambda_g \phi_g$ in the above equation with $\eta_g = \sum_{k \in F} \nu^*_{gk} q_{kg}$ where F is the set of features for which the fold changes are fixed apriori to ν^*_{gk}, and restricting i to the set $A_1 - F$ in the above derivation. Notice that there is an uncountable number of values (non-negative real values) the fold changes of features in the constraint set F can take. They will impose a particular value of η_g, and conditioned

on this value, the fold changes the rest of the features can take in group g so that a GLM achieves unbiased inference is unique.

The conclusion of theorem 1.4 is an unfortunate result as it says that to obtain unbiased inference across all features, the feature set must behave in a unique fashion, and therefore appears unlikely to occur in practice. Notice that fold-change ν^*_{gi} can never be < 0. Thus, a feasible solution need not exist for arbitrary parameter values of $\eta_g = \Lambda_g \phi_g$ implying that unbiased inference may not always be possible. It is also interesting to note here that unless the fold change of total feature content in group g (Λ_g) is somehow maintained the same across conditions despite contaminants present at proportion ϕ_g, achieving unbiased inference with normalization techniques based on the total sum is not possible. Uniquely expressed features are a major source of compositional bias and their sufficiently high expression can effectively wash out the signal. In metagenomic surveys, it is often the case that a large number of features are often observed with a positive count in very few samples. Although this does not necessarily mean they are actually present in only a few observations, we can expect this to be the case with samples arising from diverse ecosystems.

In summary, strict unbiased inference with sequencing data based on total sum based normalization approaches (like RPKM, rarification etc.,) may or may not be possible depending on the underlying value of η_g; when possible, it can only occur under a unique set of fold changes. In practice, RNAseq experiments are performed across diverse tissues of various origins, and metagenomic surveys are constantly carried out across ecosystems. Thus, unbiased-ness in inference need not hold. Is there a solution?

2 On *in silico* compositional corrections and the inability of ERCC spike-in protocol to overcome compositional bias

We need to find a transformation of our realized counts from sequencing such that we decouple every feature's fold change from everything else they occur along with. Consider the following strategy: suppose we know that some feature k is unchanged across conditions. From 2, we see that for any feature i, $q_{gi} = \Lambda_g^{-1} \nu_{gi} q_{1i}$. Because the fold change for the unperturbed feature $\nu_{gk} = 1$ for all groups g, we obtain $q_{gk} = \Lambda_g^{-1} q_{1k}$. This is a boon because if we calculate the transformation (for example, through the δ method) $\log \frac{E[Y_{gji}]}{E[Y_{gjk}]} = \log \frac{q_{gi}}{q_{gk}} = \log \frac{\Lambda_g^{-1} \nu_{gi} q_{1i}}{\Lambda_g^{-1} q_{1k}} = \mu_i + \alpha_{gi}$, where with appropriate side conditions on the contrasts, the intercept estimates $\mu_i = (\log q_{1i} - \log q_{1k})$. Our contrast variable then estimates $\alpha_{gi} = \log \nu_{gi}$, which is 0 only under the null $\nu_{gi} = 1$. Thus, roughly the traditional idea of "dividing by a feature that does not change across conditions" automatically corrects for compositionality induced through sequencing technology. Notice that we do not necessarily need the internal control feature to have the same internal concentration across conditions. As long as we know their sample-wise absolute concentrations, their fold changes across conditions are also known, and these simply enter the above formulation as known constants that simply offset the linear models. (That is, we can write: $q_{gk} = \Lambda_g^{-1} \hat{\nu}_{gk} q_{1k}$, where $\hat{\nu}_{gk}$ is the now known fold change associated with the feature in group g.) These insights bring us to the following two questions:

The utility of spike-in normalization If all we need is a feature that is expressed at known abundances across conditions, why not inject it ourselves at the time of sequencing? Two potential techniques exist in the experimental literaure, one of which cannot protect us against compositional bias. In the ERCC spike-in protocol [1], widely used in various bulk

and some single cell RNAseq studies [2], a fixed amount of total RNA extract is obtained, and subsequently suspended in solution along with known concentrations of a chosen control feature (spike-ins). Because this procedure adds the spike-ins to the extract, an already compositional source, our inferences are limited to questions on relative abundances; a statement about differences in absolute abundances cannot be made unless the samples themselves behave according to the narrow conditions established in the previous section. An alternative, more effective strategy is to add known concentrations of barcodes/spike-in to the entire sample's suspension [3]. This problem has also been noted by Stegle et al., in the context of designing scRNAseq experiments [4].

The utility of reference based scaling techniques Several normalization techniques for transcriptome analyses assume that most features are unchanged across conditions, and use some measure of central tendency ("reference") to scale the counts. For example, if one assumes that most features are unchanged ($\nu_{gi} = 1$ for most i in equation 2), averaging over the ratios of proportions in two samples can yield a robust estimate of total feature content Λ_g. Indeed, this is the strategy behind Robinson and Oshlack's TMM normalization technique [5]. A similar line of attack is to consider the ratios of a group's feature counts to their respective grand means across groups; if most features remain a constant across experimental groups, we expect a majority of these ratios to reflect the relative levels of internal feature content for a group. This is roughly the idea behind Anders and Huber's DESeq scale factor [6].

From the perspective of this work, it is only natural to ask how these fair in correcting for compositional bias. Furthermore, highly focused efforts have been geared towards the development of hefty differential expression analysis tools like that of Limma/DESeq/edgeR which additionally exploit such assumptions in the (empirical Bayes) estimation procedure through a prior on the fold changes that are centered at 0. We address these questions using empirical simulations next.

Compositional bias is dramatic with total sum / proportion based normalization approaches (as expected), and reference strategies are sensitive to the fold change distribution We now perform a simulation based analysis of compositional correction achieved by several scaling normalization techniques, in combination with several testing toolkits. We note here a fundamental problem associated with many simulation based benchmarking pipelines for differential abundance: simulating independent Poisson/Negative binomial features to generate count data across groups does not correctly incorporate the multinomial nature of sequencing. This has an adverse effect of not injecting compositional bias into the generated count data, which ultimately leads to very poor benchmarking results. As observed below, a simple re-normalization, followed by a multinomial sampling procedure as shown here overcomes this issue. Simulating feature counts for a sample independently (ex: as Poissons or Negative Binomials) does not accurately reflect sequencing count data, as the proportional nature of count generation is not made explicit in such a process. Figure 1 illustrates our simulation strategy. Given the set of control proportions q_{1i} for features $i = 1 \ldots p$, and the fraction of features that are perturbed across the two conditions $(1-\pi)$, we sample the set of true log fold changes ($\log \nu_{gi}$) from a fold change distribution for the random $(1-\pi)$ fraction of features that have been chosen to be perturbed. The fold change distribution is a two-parameter distribution chosen either as a two-parameter Uniform or a Gaussian. Based on the expressions from eqn. 3, the target proportions were then obtained as $q_{gi} = \frac{\nu_{gi} q_{1i}}{\sum_k \nu_{gk} q_{1k}}$. Conditioned on the total number of sequencing reads τ, the sequencing output Y_{gj} for all i were obtained as a multinomial with proportions vector $q_{g\cdot} = [|q_{gi}|]_{i=1}^{p}$. We set

the control proportions $q_{1\cdot}$ from various experimental datasets. With this setup, we can vary π, and the two parameters of the fold change distribution, and ask, how various normalization and testing procedures compare in terms of their performance.

With the above set up, we do not strictly enforce constant average total feature abundance across simulated cases and controls. We would like to keep the parameter variations sufficiently general that this condition roughly holds under some settings, while letting us appreciate the relative merits of reference normalization strategies under others.

In summary, for a given set of control proportions, we vary i) the fraction of features that change across conditions, ii) the shape, iii) mean and iv) variance of the fold change distribution that underlies the perturbation of features in the case-group, v) normalization approach and vi) testing technique. We also varied the control proportions themselves from various experimental datasets, and our results were similar. Our simulations are fairly general and should allow us to robustly characterize the performance of the current normalization and differential expression analysis practices in genomics.

Total Sum (RPKM/FPKM/CPM/Rarification etc.) We first analyze the total count based normalization approach (Figures 2, 3). Figure 2 plots the performance measures of edgeR for a uniform fold change distribution after total sum normalization. Sensitivity values never go beyond 65%, and heavy false positive rates are incurred even when 95% of the features remain unchanged across conditions. Figure 3 shows the performance under the Gaussian fold change distribution. In contrast to the uniform case above, we find sensitivities going up to 85%, but false positives are also accrued at higher rates. It would appear that higher variances and means lead to better performance, but as supplementary figure 6 shows, many of these truly significant features were called significant for the wrong reason: wrong signs of fold changes. Higher means and variances of fold change distributions are therefore conditions that lead to heavily confounded inference under proportion based normalization strategies. These results were similar across testing platforms, and across testing techniques (related files can be obtained from the first author.)

Reference Strategies (TMM/DESeq/Median) Figures 4 and 5 demonstrate the performance of TMM normalization, a reference based normalization strategy. In contrast to the above total sum-based normalization, the false positive rates with TMM were maintained low, if not at zero, for a variety of parameter settings. At higher fold change distribution means and variances, they also lead to wrong reconstruction of fold change signs but with a highly desirable twist: as long as the fraction of perturbed features across conditions is small, the fold change distribution is correctly centered throughout the abundance distribution except for those features with very low abundances leading to very low false positive rates (supplementary figure 6). For all normalization techniques, as the amount of features that change across conditions increases, false positive rates increase.

3 When can we hope to reconstruct X^0 from Y with non-spike-in compositional correction tools?

We can close this discussion by finally asking when in silico compositional correction is guaranteed to reconstruct true absolute abundances (X^0 in fig. 3 main text) within a sample. Consider a sequence of unknown number of (m) technical

steps on a sample j from group g, each inducing an arbitrary transformation on the intermediate abundance vectors:

$$X^0_{gj.} \xrightarrow{f_{j0}(X^0_{gj.})} X^1_{gj.} \xrightarrow{f_{j1}(X^1_{gj.})} X^2_{gj.} \longrightarrow \ldots \xrightarrow{f_{j,m-1}(X^{m-1}_{gj.})} X^m_{gj.} = X_{gj.} \text{in Fig. 3} \tag{6}$$

We will use 0 superscripted notations for those variables and parameters that correspond to the true internal state X^0. In the absence of any other information (like that of internal control features), we only get to reconstruct the compositional factors for X from Y. As long as this is proportional to the factor that take us from Y^0 to X^0, we can reconstruct X^0. The two need not be exactly equal as only relative scalings matter. Then from eqn. 2, this necessary condition implies:

$$v_{gi}\frac{q_{gi}}{q_{1i}} \propto v^0_{gi}\frac{q^0_{gi}}{q^0_{1i}}$$

$$\text{or } \left(\frac{v_{gi}}{v^0_{gi}}\right)\frac{q_{gi}}{q^0_{gi}} \propto \frac{q_{1i}}{q^0_{1i}}$$

$$\implies \frac{q_{gi}}{q^0_{gi}} \propto \frac{q_{1i}}{q^0_{1i}}$$

Now, the net perturbation experienced by feature i after the entire experiment can be written following eqn. 2 as: $a_{gi} = \lambda_g \frac{q_{gi}}{q^0_{gi}}$. Similarly, we can write an expression for the technical biases impacting reference group/sample: $a_{1i} = \lambda_1 \frac{q_{1i}}{q^0_{1i}}$. We can then write from eqn. 3 that $\frac{q_{gi}}{q^0_{gi}} = \frac{a_{gi}}{\lambda_g u_g + a_g^T q_g^0}$, where u_g represents the unique features introduced in X (possibly due to contamination) compared to X^0, and $a = [|\ldots a_{gi}\ldots|]$, the vector of perturbations introduced by the technical biases on features in X^0. Then, the above condition implies:

$$\frac{a_{gi}}{\lambda_g u_g + a^T_{g.}.q^0_{g.}} \propto \frac{a_{1i}}{\lambda_1 u_1 + a^T_{1.}.q^0_{1.}}$$

$$\text{or } \lambda_g u_g + a^T_{g.}.q^0_{g.} \propto \lambda_1 u_{1.} + a^T_{1.}.q^0_{1.} \tag{7}$$

which is also satisfied under the very specific condition when all elements in a_g are the same, $u_g = 0$, and when all elements in $a_{1.}$ are the same, and $u_1 = 0$. Notice that this condition is independent of the choice of i.

Thus, we recover a slightly more general condition than the often cited assumption on scale normalization techniques [7, 8]. We not only want the technical biases to affect all the features the same way within a sample, but if any contamination is introduced we want those biases to also behave appropriately according to the equation above. If contamination is effectively zero, then the condition is the same as those underlying scale normalization techniques.

Notice that the above simple result also suggests that if non-negligible levels of contamination happen to be introduced when going from $X^0 \to X$ in such a way that the above condition is not satisfied (which is likely to happen), we basically lose the ability to reconstruct X^0 with existing methods. In-silico post-processing of sequencing count data for contaminants (for example, by excluding reads mapping to potential cotaminant reference sequences) cannot help because they have already caused information loss by competing with other native features for being sequenced.

4 Pseudocounting with sparse datasets

In the main text, we illustrated how deriving scale factors by adding pseudocounts to sparse datasets only reflects the pseudocount's value and can be systematically predicted by sample depths/feature presence in a dataset. We mentioned that subplots (A) and (B) in main text **Fig. 5** are not sensitive the exact value of pseuodocount added. We re-generate the same plot in **Fig. 7** here for a pseudocount of 10^{-7} added to the original count data.

5 Simulation comparisons and group-wise integrity in the compositional scales reconstructed

In the main text, we compared a few approaches with our proposed technique (Wrench). In the next two subsections, we catalogue 1) all the simulation results corresponding to how well the compared techniques worked in reconstructing scales for sparse count data and 2) illustrate how many of the compared techniques showed experimental group-wise integrity in the scales they reconstructed, a result that indicates the importance of compositional correction in general practice.

In all these figures, the main text estimators $W_0 ... W_3$, are mentioned as "wrench.hurdle", "wrench.wmmean", "wrench.zadjF.s2 [illegible] respectively.

5.1 Simulation comparisons

In the main text, we demonstrated simulation performances with various fraction of features perturbed across two groups, at various sequencing depths with proportions from the control group derived from the mouse microbiome dataset. Because compositional biases lead to different sparsity fingerprints depending on the underlying proportions, here we also derive the control proportions from the diarrheal and lung microbiome datasets, and show in **Figs. 8** that the performances are robust to control proportions. In Fig. 9, we also show how Wrench compares with the centered logarithmic transform with mouse microbiome proportions. Behavior was similar across dataset proportions, with CLR not yielding better differential calls and poor group-wise reconstructions.

5.1.1 Rough Simulations that illustrate the point

In the Results section we formalized compositional bias and described the central idea behind looking at ratios of proportions of samples to some reference. We also illustrated how with sparse count data obtained in metagenomics, TMM/DESeq estimation techniques suffer as the feature-wise estimates are either not well-defined or mostly assume zero values. Scran, developed for sparse but high coverage scRNAseq data, aimed to overcome these limitations by noting that summed feature-wise estimates across any set of samples are linear in the linear technical biases associated with the samples. The idea was then to solve a linear regression system by constructing simulated, albeit highly correlated, observations by summing counts of various subsets of samples in a dataset. This technique fails to reconstruct scales for undersampled count data / at heavy sparsity (see below). In metagenomic analysis, CSS was developed as a scale normalization technique by summing the counts of the relatively invariant part of the truncated (positive-only) count distribution for every sample upto a quantile that shows substantial variations in the dataset.

With a simple simulation (described in detail in the Methods section), we shall illustrate these caveats and where the current state of the art stands. Roughly, we simulated two experimental groups with roughly $54K$ features, and let 35% of features change across conditions. As can be seen from the **table 2**, and **Fig. 7**, in the maint text, the following observations formed the theme of our results section: TMM/CSS, predominantly because they focus on positive-valued observations only, are restricted in the range of scales they can reconstruct in general. 2) Scran can yield accurate estimators at very large sequencing depths when high feature-wise coverages are achieved. Unfortunately, this behavior is highly dependent on the underlying feature proportions and their diversity. 3) We have argued that Wrench estimators

are better alternatives for under-sampled data, and still offer robust protection at higher coverages.

5.2 Organization of reconstructed scales with respect to phenotype

Figs. 10, 12, 13 and 14 illustrate the organization of reconstructed scales with respect to phenotype. In each case, we also plot the average of the positive valued raw proportion ratios with respect to the reference, which was chosen as the average proportion vector across samples in each dataset.

For completeness, we also attach similar results from all the 11 organs of the rat body map dataset here. We noted that the rat body map samples also showed systematic tissue-specific global deviations in the expressed features' fold change distribution. We include this analysis of low sparsity samples for completeness. **Fig. 15** shows this result and the general behavior of compositional scales across various methods compared and a few related statistics of the dataset. Given that these samples arise from a well designed series of experiments, and that the similarity in the scales within and across related tissues, and across normalization methods, is striking, the observed trend in the reconstructed scales could indeed reflect underlying true compositional differences for the most part. TMM and CSS ascribe substantially deviated scales to muscle, heart and liver tissues, when compared to Scran and Wrench estimators. This effect may be due to the truncated estimation strategy which biases the scales for a relatively fewer but highly expressed genes in these tissues. Nevertheless, these results indicate potentially heavy compositional bias injected into downstream differential abundance analysis that compare tissues of different types. These results suggest that compositional bias can be costly not only in metagenomics, but even in common bulk-RNAseq studies.

6 Possible sources of sparsity in metagenomic and single cell RNAseq data

Based on our simulation results, and existing observations on experimental data, compositional bias could be a potential source of sparsity in high-resolution surveys like single cell RNAseq and metagenomics (**Fig. 16**). In metagenomics, zeroes in the count distributions of the surveyed species could occur due to a variety of reasons: first, low average abundances of microbes can induce zeroes in the survey data as a result of the Poisson process of sampling reads when the sequencing coverage is insufficient. Second, microbes being dynamic, species interactions can result in fluctuations of species abundances causing cycles of high to low abundances; given that cross-sectional data are not necessarily aligned in time with respect to the dynamics, such sampling induced zeroes can occur. Third, very closely related to the previous point, but subtly different is the compositional nature of the observed counts, where highly expressed species can sequester sequencing reads, such that the normalized multinomial sampling probabilities favors their own expression. This leads to reduced/zero-read generation from other low abundant species. Finally, an observed zero could reflect the true absence of species in a sample.

Given that pooling across single cell transcriptomes reproducibly recapitulates bulk RNAseq count distributions [9], technical differences in the bulk and single cell RNAseq experiments need not be the sole, systematic driving factors of sparsity in single cell RNAseq count data. We resort to the well-documented biophysical observations on gene expression: transcriptional bursting (also noted by Lun and colleagues [8]). This is a fundamental, highly conserved phenomenon across genes, across organisms [10, 11, 12], where cycles of periods of inactivity, followed by burst phases in transcript expression arise that ultimately leads to a dynamic, pulsed expression of gene transcripts. When prob-

ing gene expression in single cells – asynchronous in their expression state – one expects tremendous variations in the recorded gene counts owing to compositional effects. As with metagenomics, indeed, a recorded zero could reflect a true absence in the expression for a given gene. We can also relate to some previous findings on sample-wise feature detection rates in single cell RNAseq to compositional bias. Hicks and Irizarry [13] find the median expression values of positive counts in samples of a dataset to increase with increasing sparsity. Indeed, this was shown as a direct effect arising from compositional bias in Fig. 6 in the main text. Results of Finak et al., 2015 indicate how detection rates contribute to much of the variation in single cell count data, and the authos recommend controlling for it in the mean models.

7 Derivation of weights used in models section in the main text.

Setting $\phi_{0i} = e^{\sigma^2_{0i}/2}$, and $\gamma_{0g} = e^{\eta^2_{0g}/2}$, we have:

$$\begin{aligned} Var_\theta(E(Y_{gji}|\theta_{gji})) &= Var_\theta((1-\pi_{gji})\theta_{gji}\tau_{gj}q_{0i}\phi_{0i}) \\ &= ((1-\pi_{gji})\tau_{gj}q_{0i}\phi_{0i})^2 \underbrace{(\gamma^2_{0g}-1)\gamma^2_{0g}\zeta^2_{0g}}_{\text{group specific contribution}} \end{aligned} \tag{8}$$

Now, if we let Z to be an indicator random variable denoting whether a feature was zero or positive:

$$\begin{aligned} Var(Y_{gji}|\theta_{gji}) &= E_Z(Var(Y_{gji}|\theta_{gji}, Z)) + Var_Z(E(Y_{gji}|\theta_{gji}, Z)) \\ &= (1-\pi_{gji})\left(\theta_{gji}\tau_{gj}\phi_{0i}q_{0i}\right)^2\left[\pi_{gji} + (\phi^2_{0i}-1)\right] \end{aligned} \tag{9}$$

Similarly,

$$\begin{aligned} E(\theta^2_{gji}) &= Var(\theta_{gji}) + E(\theta^2_{gji}) \\ &= \left(\gamma^2_{0g}-1\right)\gamma^2_{0g}\zeta^2_{0g} + (\zeta_{0g}\gamma_{0g})^2 \\ &= (\zeta_{0g}\gamma_{0g})^2\gamma^2_{0g} \end{aligned} \tag{10}$$

Together, eqns. 8 and 9 lead to:

$$E(Var(Y_{gji}|\theta_{gji})) = (1-\pi_{gji})\left[\pi_{gji} + (\phi^2_{0i}-1)\right](q_{0i}\tau_{gj}\phi_{0i})^2\left(\gamma^2_{0g}\zeta_{0g}\right)^2 \tag{11}$$

Eqns. 8 and 11 then imply:

$$\begin{aligned} Var(Y_{gji}) &= (1-\pi_{gji})(q_{0i}\tau_{gj}\phi_{0i})^2\left[\pi_{gji} + \phi^2_{0i}\gamma^2_{0g} - 1\right]\gamma^2_{0_g}\zeta^2_{0g} \\ &\propto (1-\pi_{gji})(q_{0i}\tau_{gj}\phi_{0i})^2\left[\pi_{gji} + \phi^2_{0i}\gamma^2_{0g} - 1\right] \end{aligned} \tag{12}$$

The variances for the adjusted ratios then follows from straightforward calculations, the inverse of which take the weight forms shown in in the model section of the main text.

8 Can we extend DESeq style factors for sparse count data, with a hurdle overlay?

One disadvantage of the model proposed in the main text is that the distributional form of the reference q^*_i, which was defined as the average proportion of feature i across the entire dataset does not have a convenient distributional form, sample-wise expressions are determined by the log-normal as assumed. However, products and quotients of log-normal

variantes can be easily characterized, and this made us wonder if it was possible to use DESeq-style factors for estimation purposes. Below, we will consider two cases: 1) when all features can be assumed to be expressed with a positive count in all samples and 2) when feature expression patterns are sparse, as considered in the main text. In the latter case, we specifically asked how computing DESeq style factors on the positive part of data alone would behave under model assumptions. Both derivations will prove to be interesting in terms of future directions to the body of work presented in this paper.

8.1 Case 1: A normalization strategy when all features are expressed in all samples

Ignoring group information, we shall use $i = 1 \dots p$ and $j = 1 \dots n$ to index features and samples/observations respectively. For any sample j, Y_{ij} indicates its count of feature i and $\tau_j = \sum_i Y_{ij}$ indicates its total feature count. We will make similar classical assumptions for feature-wise count data, as was made for the postive part of the hurdle model in the main text. In particular, we will start of by assuming that feature-wise count distributions follow a log-normal distribution, and observe how this leads to a DESeq-like estimator:

$$\begin{aligned} Y_{ij} &\sim \Lambda_j^{-1} \cdot \tau_j q_i^* \cdot LN(0, \sigma_i^2), \;\; i = 1 \dots p, \;\; j = 1 \dots n \\ &\equiv \mu_{ij} \cdot LN(0, \sigma_i^2) \end{aligned}$$

Then:

$$\begin{aligned} &\prod_j Y_{ij} \sim \left(\prod_j \mu_{ij}\right) LN(0, n\sigma_i^2) \\ &\left[\prod_j Y_{ij}\right]^{\frac{1}{n}} \sim \left(\prod_j \mu_{ij}\right)^{1/n} LN(0, \sigma_i^2) \\ &\implies d_{ij} = \frac{Y_{ij}}{\left(\prod_j Y_{ij}\right)^{\frac{1}{n}}} \sim \frac{\mu_{ij}}{\left(\prod_j \mu_{ij}\right)^{\frac{1}{n}}} \cdot LN(0, \sigma_i^2) \\ &= \frac{\Lambda_j^{-1}}{\left(\prod_j \Lambda_j^{-1}\right)^{\frac{1}{n}}} \cdot \frac{\tau_j}{\left(\prod_j \tau_j\right)^{\frac{1}{n}}} \cdot LN(0, \sigma_i^2) \\ &= k_\Lambda \Lambda_j^{-1} \cdot k_\tau \tau_j \cdot LN(0, \sigma_i^2) \end{aligned} \tag{13}$$

in which we have collected the constant denominator (independent of j) terms of Λ_j and τ_j factor separately into two k terms with corresponding subscripts. Now, $\tilde{d}_{ij} = \frac{d_{ij}}{k_\tau \tau_j} \sim k_\Lambda \Lambda_j^{-1} \cdot LN(0, \sigma_i^2)$, with expectation given by $k_\Lambda \Lambda_j^{-1} e^{\sigma_i^2/2} \propto \Lambda_j^{-1} e^{\sigma_i^2/2}$. Thus if a median fraction of features do not change across conditions, then on average,

$$median_i \; \frac{\tilde{d}_{ij}}{e^{\sigma_i^2/2}} \propto \Lambda_j^{-1} \tag{14}$$

serves as an estimator of Λ_j^{-1}. This is simply DESeq normalization factors altered by feature-wise variances. Extensions to this basic approach by overlaying priors - as illustrated in the main text - can be conveniently made.

8.2 Case 2: DESeq style estimators for sparse data.

Ignoring group information, we shall use $i = 1 \dots p$ and $j = 1 \dots n$ to index features and samples/observations respectively. For any sample j, Y_{ij} indicates its count of feature i and $\tau_j = \sum_i Y_{ij}$ indicates its total feature count. Further, we denote by $\omega_j = \{i : q_{ij} > 0\}$, the set of features expressed in the sample, and by $\xi_i = \{k : q_{ik} > 0\}$, the set of samples that

express feature i. We will denote by $\xi_{ij} = \xi_i - \{j\} = \{k, k \neq j : q_{ik} > 0\}$, the set of samples that express feature i excluding sample j. Notice $|\xi_{ij}| = |\xi_i| - 1$ for all j. Based on eqn. 2, and adjusting for sample depth, we can write: $E[\log Y_{ij} | Y_{ij} > 0] = \log\left(\tau_j \Lambda_j^{-1} \nu_{1i} q_i^*\right) \equiv \log \mu_{ij}$. Our goal is to estimate Λ_j, the compositional scale factor for sample j that we want to estimate. If we start by assuming a marginally independent hurdle log-normal model for the features:

$$Y_{ij} \sim \pi_{ij}\delta_0 + (1-\pi_{ij}) \cdot (\mu_{ij}) \cdot LN(0, \sigma_i^2) \tag{15}$$

we can show (**supplementary section 8.2.1**) that this imposes the following distribution on $h_{ij}|\xi_{ij} = \frac{Y_{ij}}{\left(\prod_{k\in\xi_{ij}} Y_{ik}\right)^{\frac{1}{\xi_{ij}}}}$, which are simply DESeq style scale factors computed only on the positive part of the data,

$$h_{ij}|\xi_{ij} \equiv \frac{Y_{ij}}{\left(\prod_{k\in\xi_{ij}} Y_{ik}\right)^{\frac{1}{|\xi_{ij}|}}} \sim \pi_{ij}\delta_0 + (1-\pi_{ij}) \cdot \overline{\mu_{ij}} \cdot LN\left(0, \sigma_i^2\left[1 + \frac{1}{|\xi_i| - 1}\right]\right) \tag{16}$$

where $\overline{\tau_{ij}} = \frac{\tau_j}{\left(\prod_{k\in\xi_{ij}} \tau_k\right)^{\frac{1}{\xi_{ij}}}}$, and $\overline{\nu_{ij}} = \frac{\nu_{ij}}{\left(\prod_{k\in\xi_{ij}} \nu_{ik}\right)^{\frac{1}{\xi_{ij}}}}$. It is therefore clear that the h_{ij} s computed from data are not identically distributed across all the features even within a sample. Indeed, each has a distinct expectation and variance determined significantly by the samples that exhibit the respective feature expression:

$$E[h_{ij}|\xi_{ij}, \overline{\mu_{ij}}] = (1-\pi_{ij}) \cdot \overline{\mu_{ij}} \cdot \underbrace{e^{\sigma_i^2/2\left[1+\frac{1}{|\xi_i|-1}\right]}}_{\equiv \phi_i}$$

$$= (1-\pi_{ij}) \cdot \left[\frac{\Lambda_j^{-1}}{\left(\prod_{k\in\xi_{ij}} \Lambda_k^{-1}\right)^{\frac{1}{|\xi_i|-1}}} \cdot \underbrace{\frac{\tau_j}{\left(\prod_{k\in\xi_{ij}} t_k\right)^{\frac{1}{|\xi_i|-1}}}}_{\equiv\overline{\tau_{ij}}} \cdot \underbrace{\frac{\nu_{ij}}{\left(\prod_{k\in\xi_{ij}} \nu_{ik}\right)^{\frac{1}{|\xi_i|-1}}}}_{\equiv\overline{\nu_{ij}}}\right] \cdot \phi_i$$

We can further write:

$$E[\tilde{h}_{ij}|\xi_{ij}, \pi_{ij}, \overline{\tau_{ij}}, \nu_{ij}, \phi_i] = \frac{1}{(1-\pi_{ij}) \cdot \overline{\tau_{ij}} \cdot \overline{\nu_{ij}} \cdot \phi_i} E[h_{ij}] = \Lambda_j^{-1}\left(\prod_{k\in\xi_{ij}} \Lambda_k\right)^{\frac{1}{|\xi_i|-1}} \tag{17}$$

which is log-linear in the log-compositional scales of all the samples used to compute h_{ij}. Under the assumption that $\overline{\nu_{ij}} = 1$ for most i and j (for example, when features are not differentially expressed), and after estimating $E[h_{ij}]$ with h_{ij}, and the π_{ij}s from logistic regression, the left-hand side of the above equation is available. With this set of estimates, in principle, one can take an optimization route to estimating compositional scales with linear regressions. We return to this point in the next subsection, but first consider an additional simplifying assumption to estimate compositional scales. If the second term on the right-hand side can be considered to be roughly similar across i and j, it serves to only scale the equations by a constant factor. For example, when most features are expressed in all samples as usually is the case with RNAseq datasets, it is simply the geometric mean of compositional scales across samples.) Because only the relative scales matter, this term can be ignored. Under the limit that all surveyed features are present in all samples, and when feature-wise variances are similar, the above factor is indeed the DESeq scale factor. Roughly, one can choose:

$$\overline{\Lambda_j^{-1}} = \text{median}_{i\in w_j} \widehat{E[\tilde{h}_{ij}]}^{-1} \tag{18}$$

as the compositional scale for sample j.

Moderated estimation As in the main text, here too, one can proceed in the same fashion to regularize estimators. Weighted estimation strategy can then proceed similarly as with the model developed in the main text.

The limited utility of an estimation strategy based on linear regressions in sparse data.

Based on eqn. 17, we remarked that $\log E[\tilde{h}_{ij}]$ are linear in $\log \Lambda_j, j \in \xi_{ij}$, which lead us directly to the regression route. Here is a complication: straightforward regression equations built this way for feature i across different samples j do not reflect equations arising from independent observations as they share $|\xi_i| - 2$ count ratios (ref eqn. 16. $|\xi_i|$ is the number of samples expressing feature i), thus not satisfying one of the fundamental assumptions of classical linear regression. But it is also easy to see that that the quantitites are roughly independent across i in different samples j (within samples, the features are still tied together by a multinomial due to sequencing technology); so, if we are able to construct our equations in such a way that it includes one i for each sample j, we get a regression system built for roughly independent data. But this is not as simple as picking a random i for each sample j as not all features are expressed with a positive count in each sample. For instance, the median number of samples in which features are expressed in the diarrheal microbiome study (methods) (with a roughly 1000 samples) was 8. For the mouse and lung study, these numbers were 4 and 3 respectively. But this also does not necessarily mean that no solutions exist to this problem: the number of features is large - on the order of tens of thousands. We therefore cast this as an instance of a matching problem, a classic problem studied in computer science for which efficient polynomial time algorithms exist (illustrated in **Fig. 17**). To make sure that the resulting system of equations are full rank, borrowing a simple useful idea from Scran, we append a full rank n-dimensional system of equations, with a corresponding response derived in eqn. 18. That is, each column votes for its median estimate. We repeat the procedure for two hundred matching solutions (or less depending on how many are found), and then take the median of the solutions as our final compositional scale estimates. While in simulated data, we did not find a major difference in the resulting estimators when compared to that of those arising from eqn. 18, in the metagenomic datasets we worked with, this approach lead to unreliable estimates: the high sparsity simply was too unweildy to result in many different stable matchings to be useful. We have experimented with different weighting strategies (both at the level of solving the matching problem and at the level of solving the regression system), for instance using inverse variances of $\log \tilde{h}_{ij}$s and bootstrapped estimates of variances of the medians. We leave the further development of these ideas to future work.

8.2.1 Derivation of Equation. 16 above.

The key trick throughout the main text is conditioning on ξ_{ij} and utilizing the transformation results of lognormal random variables. Because we have conditioned on the set ξ_{ij}, the denominator of h_{ij} is always positive. As a result, the hurdle parameters for h_{ij} only reflect the probability that $Y_{ij} = 0$ and not a function of the $Y_{ik}, k \in \xi_{ij}$. Also, the definition of h_{ij} in equation. 16, by excluding y_{ij}, also avoids the case of not offering well-defined ratios when $y_{ij} = 0$.

$$\prod_{k\in\xi_{ij}} Y_{ik} \sim \left(\prod_{k\in\xi_{ij}} \mu_{ik}\right)\cdot LN\left(0, \sum_{k\in\xi_{ij}} \sigma_i^2\right)$$

$$\sim \left(\prod_{k\in\xi_{ij}} \mu_{ik}\right)\cdot LN\left(0, \underbrace{|\xi_{ij}|}_{=|\xi_i|-1\forall\ j} \ \sigma_i^2\right)$$

So:

$$\left[\prod_{k\in\xi_{ij}} Y_{ik}\right]^{1/|\xi_{ij}|} \sim \left(\prod_{k\in\xi_{ij}} \mu_{ik}\right)^{1/|\xi_{ij}|} \cdot LN\left(0, \frac{1}{|\xi_{ij}|^2}|\xi_{ij}|\sigma_i^2\right)$$

$$\sim \left(\prod_{k\in\xi_{ij}} \mu_{ik}\right)^{1/|\xi_{ij}|} \cdot LN\left(0, \frac{1}{|\xi_{ij}|}\sigma_i^2\right)$$

With $h_{ij} = \frac{Y_{ij}}{\left[\prod_{k\in\xi_{ij}} Y_{ik}\right]^{1/|\xi_{ij}|}}$, where all $Y_{ik} > 0, k \in \xi_{ij}$, h_{ij} is zero $\iff$ $Y_{ij} = 0$. Thus it inherits the same hurdle probability as that of the marginal of Y_{ij}. Conditioned on the event that $Y_{ij} > 0$, h_{ij} reduces to the ratio of two independent log-normals, and therefore follows:

$$\frac{Y_{ij}}{\left[\prod_{k\in\xi_{ij}} Y_{ik}\right]^{1/|\xi_{ij}|}}|Y_{ij} > 0 \sim \frac{\mu_{ij}}{\left(\prod_{k\in\xi_{ij}} \mu_{ik}\right)^{1/|\xi_i|-1}} \cdot LN\left(0, \sigma_i^2 + \frac{1}{|\xi_i|-1}\sigma_i^2\right)$$

We then arrive at eqn. 16 above.

8.2.2 Variance estimators for the aforementioned DESeq-style factors.

Suppose h_{ij} follows a hurdle log-normal model as below.

$$h_{ij} \sim \pi_{ij}\delta_0 + (1-\pi_{ij})\cdot(\overline{\mu_{ij}})\cdot LN(0,\sigma_i^2) \tag{19}$$

Some conditional expectations and variances are given below, which are then used to build slightly more general expectations and variances:

$$E[h_{ij}|\xi_{ij}, \overline{\mu_{ij}}, Z_{ij} = 1] = 0$$

$$Var[h_{ij}|\xi_{ij}, \overline{\mu_{ij}}, Z_{ij} = 1] = 0$$

$$E[h_{ij}|\xi_{ij}, \overline{\mu_{ij}}, Z_{ij} = 0] = \overline{\mu_{ij}}\phi_i$$

$$Var[h_{ij}|\xi_{ij}, \overline{\mu_{ij}}, Z_{ij} = 0] = \overline{\mu_{ij}}^2\phi_i^2(\phi_i^2-1)$$

First, we notice:

$$E[h_{ij}|\xi_{ij}, \overline{\mu_{ij}}] = (1-\pi_{ij})\overline{\mu_{ij}}\phi_i \tag{20}$$

and,

$$E_Z[Var[h_{ij}|\xi_{ij}, \overline{\mu_{ij}}, Z_{ij}] = (1-\pi_{ij})\overline{\mu_{ij}}^2\phi_i^2(\phi_i^2-1), \text{and}$$

$$Var_Z[E[h_{ij}|\xi_{ij}, \overline{\mu_{ij}}, Z_{ij}]] = E_Z[E[h_{ij}|\xi_{ij}, \overline{\mu_{ij}}, Z_{ij}]^2] - E_Z[E[h_{ij}|\xi_{ij}, \overline{\mu_{ij}}, Z_{ij}]]^2$$

$$= \left[\pi_{ij}\cdot 0 + (1-\pi_{ij})\overline{\mu_{ij}}^2\phi_i^2\right] - [\pi_{ij}0 + (1-\pi_{ij})\mu_{ij}\phi_i]^2$$

$$= (1-\pi_{ij})\overline{\mu_{ij}^2}\phi_i^2\,[1-(1-\pi_{ij})]$$

$$= \pi_{ij}(1-\pi_{ij})\overline{\mu_{ij}}^2\phi_i^2$$

which together lead, through the conditional variance formula, to:

$$Var(h_{ij}|\xi_{ij},\overline{\mu_{ij}}) = (1-\pi_{ij})\overline{\mu_{ij}}^2\phi_i^2\left[\pi_{ij}+(\phi_i^2-1)\right] \quad (21)$$

We subsequently defined $\tilde{h}_{ij}|\xi_{ij},\mu_{ij} = \left[\frac{1}{(1-\pi_{ij})\phi_i\tau_{ij}}\right]h_{ij}$, and were interested in estimating the following expectation as it was log-linear in the logged-compositional scales (under the assumption that $\overline{v_{ij}} = 1$):

$$E[\tilde{h}_{ij}|\xi_{ij},\overline{\mu_{ij}}] = \left[\frac{1}{(1-\pi_{ij})\phi_i\overline{\tau_{ij}}}\right]E[h_{ij}|\xi_{ij},\overline{\mu_{ij}}].$$

Estimating $\overline{\mu_{ij}}$ with h_{ij}, and noting that $Var(\widehat{E[h_{ij}]}) = Var(h_{ij})/1$, we find:

$$\begin{aligned} Var(\log\widehat{E[\tilde{h}_{ij}|\overline{\mu_{ij}}]}) &= \frac{1}{\widehat{E[\tilde{h}_{ij}|\overline{\mu_{ij}}]}^2}Var[\widehat{E[\tilde{h}_{ij}|\overline{\mu_{ij}}]}] \\ &= (1-\pi_{ij})\phi_i^2\left[\pi_{ij}+(1-\pi_{ij})(\phi_i^2-1)\right] \end{aligned} \quad (22)$$

Truncated analysis Sometimes, one may wish to perform a truncated analysis by ignoring zeroes. We present the estimators for that case. From equation 16 above,

$$h_{ij}|h_{ij}>0 \sim (\overline{\mu_{ij}})\ LN\left(0,\sigma_i^2\left[1+\frac{1}{|\xi_i|-1}\right]\right) \quad (23)$$

where as before $\overline{\mu_{ij}} = \overline{\Lambda_{ij}^{-1}\tau_{ij}v_{ij}}$. Defining, $\tilde{h}^*_{ij}|h_{ij}>0 \equiv h_{ij}/\overline{v_{ij}\tau_{ij}}\phi_i$ we obtain:

$$\tilde{h}^*_{ij}|h_{ij}>0 \sim \left(\frac{\overline{\Lambda_{ij}^{-1}}}{\phi_i}\right)\ LN\left(0,\ \sigma_i^2\left[1+\frac{1}{|\xi_i|-1}\right]\right) \quad (24)$$

with $E[\tilde{h}^*_{ij}] = \overline{\Lambda_j^{-1}}$, which is log-linear in the logged compositional scales. As before, we can assume $\overline{v_{ij}} = 1$, and build regressions straight up from these equations. To address the heteroskedastic nature of the $\tilde{h}_{ij}$, we perform weighted least squares, with weights set to inverse variances of $\log E[\tilde{h}^*_{ij}]$, which can be derived as:

$$\begin{aligned} Var(\log\widehat{E[\tilde{h}^*_{ij}]}) &= \frac{1}{\widehat{E[\tilde{h}^*_{ij}]}^2}\cdot Var(\widehat{E[\tilde{h}^*_{ij}]}) \\ &= \frac{1}{\left(\tilde{h}^*_{ij}\right)^2}\cdot Var(\tilde{h}^*_{ij})/1 \\ &= \phi_i^2(\phi_i^2-1) \end{aligned} \quad (25)$$

Here, as before, $Var(h_{ij}|h_{ij}>0) = \phi_i^2(\phi_i^2-1)\overline{\mu_{ij}}^2$, where $\overline{\mu_{ij}}$ is estimated with h_{ij}.

9 Bland-Altman plots for correlation analyses

In Figs. 18, 19 and 20, we present the Bland-Altman plots for data underlying Table 3 of main text. In all these figures, the Wrench estimators $W_0...W_3$ in the main text, are mentioned as "wrench.mean", "wrench.hurdle", "wrench.wmmean", "wrench.zadjF.s2w" respectively. These are the corresponding parameter values input in the Wrench program for the estimator choice.

As mentioned in the main text, for the Tara project, the inverse of the total cell count for each sample was compared with the compositional scale factors. In the UMI and the Rat body map datasets, normalization factors for each sample

were compared with the corresponding total spike-in counts for each sample. The analysis was performed across all the methods compared. In the corresponding Bland-Altman plots, we noticed a linear trend in the deviations across all methods in at least one of these datasets. As shown in supplementary fig. 24, in the case of UMI and the Rat body map datasets, we observed unexpected linear trends in spike-in proportions (which could be thought of as compositional factors based on spike-in counts alone) as a function of sample-depth. This behavior however was not shared by the normalization factors from all the compared normalization methods, and so the deviations in the Bland-Altman plots are arising from this descrepancy/unexpected behavior from the spike-ins.

Also, as mentioned in the last results section, all normalization methods had reasonable agreements in their scale factors within phenotypes. Also notice that, in these experimental studies, unlike the Loven et al., [3] spike-in procedure, the ERCC procedure is followed where fixed quantities of spike-ins were added to extracts of source samples. Therefore, based on our discussions in supplementary section 2, we can only expect them to capture other sources of technical variation beyond compositional bias induced by differential expression.

10 Balanced and Unbalanced Designs

In Figs. 21 and 22. we present the simulation results for exploring the performance of Wrench (for comparison, TMM is presented as well) on sample size dependence and fraction of features that change across conditions.

11 Bechmarking analysis on the small scale high coverage miRNA data

In Fig. 23, we present the same benchmarking analysis as in Fig. 7 Argyropolous et al., [14] for DeSeq2, GAMLSS, Wrench normalization + EdgeR and Scran normalizaiton + EdgeR pipelines for differential abundance. The data was downloaded from authors' repository: https://bitbucket.org/chrisarg/rnaseqgamlss.

References

[1] L. Jiang, F. Schlesinger, C. A. Davis, Y. Zhang, R. Li, M. Salit, T. R. Gingeras, and B. Oliver, "Synthetic spike-in standards for RNA-seq experiments," *Genome research*, vol. 21, no. 9, pp. 1543–1551, 2011.

[2] P. Brennecke, S. Anders, J. K. Kim, A. A. Kołodziejczyk, X. Zhang, V. Proserpio, B. Baying, V. Benes, S. A. Teichmann, J. C. Marioni, and M. G. Heisler, "Accounting for technical noise in single-cell RNA-seq experiments," *Nature Methods*, vol. 10, pp. 1093–1095, Nov. 2013.

[3] J. Lovén, D. A. Orlando, A. A. Sigova, C. Y. Lin, P. B. Rahl, C. B. Burge, D. L. Levens, T. I. Lee, and R. A. Young, "Revisiting global gene expression analysis," *Cell*, vol. 151, pp. 476–482, Oct. 2012.

[4] O. Stegle, S. A. Teichmann, and J. C. Marioni, "Computational and analytical challenges in single-cell transcriptomics," *Nature Reviews Genetics*, vol. 16, pp. 133–145, Mar. 2015.

[5] M. D. Robinson and A. Oshlack, "A scaling normalization method for differential expression analysis of RNA-seq data," *Genome Biology*, vol. 11, p. R25, Mar. 2010.

[6] S. Anders and W. Huber, “Differential expression of RNA-Seq data at the gene level–the DESeq package,” *Heidelberg, Germany: European Molecular Biology Laboratory (EMBL)*, 2012.

[7] R. A. Irizarry, B. Hobbs, F. Collin, Y. D. Beazer-Barclay, K. J. Antonellis, U. Scherf, and T. P. Speed, “Exploration, normalization, and summaries of high density oligonucleotide array probe level data,” *Biostatistics*, vol. 4, no. 2, pp. 249–264, 2003.

[8] A. T. L. Lun, K. Bach, and J. C. Marioni, “Pooling across cells to normalize single-cell RNA sequencing data with many zero counts,” *Genome Biology*, vol. 17, p. 75, 2016.

[9] A. R. Wu, N. F. Neff, T. Kalisky, P. Dalerba, B. Treutlein, M. E. Rothenberg, F. M. Mburu, G. L. Mantalas, S. Sim, M. F. Clarke, and S. R. Quake, “Quantitative assessment of single-cell RNA-sequencing methods,” *Nature methods*, vol. 11, pp. 41–46, Jan. 2014.

[10] I. Golding, J. Paulsson, S. M. Zawilski, and E. C. Cox, “Real-Time Kinetics of Gene Activity in Individual Bacteria,” *Cell*, vol. 123, pp. 1025–1036, Dec. 2005.

[11] D. M. Suter, N. Molina, D. Gatfield, K. Schneider, U. Schibler, and F. Naef, “Mammalian Genes Are Transcribed with Widely Different Bursting Kinetics,” *Science*, vol. 332, pp. 472–474, Apr. 2011.

[12] S. Chong, C. Chen, H. Ge, and X. S. Xie, “Mechanism of Transcriptional Bursting in Bacteria,” *Cell*, vol. 158, pp. 314–326, July 2014.

[13] S. C. Hicks, M. Teng, and R. A. Irizarry, “On the widespread and critical impact of systematic bias and batch effects in single-cell RNA-Seq data,” *bioRxiv*, p. 025528, Sept. 2015.

[14] C. Argyropoulos, A. Etheridge, N. Sakhanenko, and D. Galas, “Modeling bias and variation in the stochastic processes of small RNA sequencing,” *Nucleic Acids Research*, vol. 45, p. e104, June 2017.

[15] Y. Yu, J. C. Fuscoe, C. Zhao, C. Guo, M. Jia, T. Qing, D. I. Bannon, L. Lancashire, W. Bao, T. Du, H. Luo, Z. Su, W. D. Jones, C. L. Moland, W. S. Branham, F. Qian, B. Ning, Y. Li, H. Hong, L. Guo, N. Mei, T. Shi, K. Y. Wang, R. D. Wolfinger, Y. Nikolsky, S. J. Walker, P. Duerksen-Hughes, C. E. Mason, W. Tong, J. Thierry-Mieg, D. Thierry-Mieg, L. Shi, and C. Wang, “A rat RNA-Seq transcriptomic BodyMap across 11 organs and 4 developmental stages,” *Nature Communications*, vol. 5, p. 3230, Feb. 2014.

[16] Z. D. Kurtz, C. L. Müller, E. R. Miraldi, D. R. Littman, M. J. Blaser, and R. A. Bonneau, “Sparse and Compositionally Robust Inference of Microbial Ecological Networks,” *PLOS Comput Biol*, vol. 11, p. e1004226, May 2015.

List of Figures

1 **Simulation strategy for evaluating current normalization and differential expression analysis toolkits for compositional correction.** (A) Simulation set up. q_1, q_g represent the control and case proportion vector of all the features. q_1 is obtained from a given experimental dataset. π represents the fraction of features that do not change across conditions. Z_g $Bernoulli(\pi)$ represents the set of indicator variables that denote if a feature is not differentially expressed. Conditioned on Z_g, the logged vector of fold changes $\log \nu$ is sampled from a two-parameter fold change distribution, with ν_{gi} set to 1 whenever Z_{gi} is 1. Here i indexes the individual entries of the vector. The sampled fold changes and control proportions are normalized to yield the case proportions. A multinomial draw for a fixed sample depth τ (20M reads) then yields the desired simulated sequencing output. The two fold change distributions, $Unif(a,b)$ and a $N(\mu,\sigma^2)$, considered in our study are shown in (B). Example simulations when 75% (i.e., $\pi = 0.75$) of the features are fixed across conditions, with the rest perturbed according to log fold changes sampled from $Normal(0,1)$ and $Unif(-4,4)$ fold change distributions respectively are shown in (C). 21

2 **Total sum based normalization, like RPKM/Rarefication, under a Uniform fold change distribution.** The figure plots various performance metrics of the edgeR package as a function of the fraction of features that remain unchanged across conditions (π), and the lower (a) and upper bounds (b) of a Uniform fold change distribution. Control proportions (q_1) were obtained from rat liver tissue of the rat bodymap [15]. Extremely high false positive rates result with higher variance and asymmetrically located fold change distributions (i.e., with positive or negative means) due to compositionality induced confounding. The results were similar across testing platforms, and for the Gaussian fold change distribution(fig. 3). 22

3 **Total sum based normalization, like RPKM/Rarefication, under a Gaussian fold change distribution.** The figure plots various performance metrics of the edgeR package as a function of the fraction of features that remain unchanged across conditions (π), and the mean (μ) and standard deviation (σ) of the Gaussian fold change distribution for the same control proportions (q_1) as in figure 5. (A) (σ, π) variations at $\mu = 0$. (B) (μ, π) variations at $\sigma = 1$. It would appear that higher fold change distribution variances and means lead to better performance, but these are also associated with higher false positive rates and as figure 6shows, large fraction of these calls had wrong signed fold changes. Higher means and variances of fold change distributions are therefore cases that lead to heavily confounded inference. The results were similar across testing techniques. 23

4 **Reference normalization (TMM/DESeq/Median) under a Uniform fold change distribution.** The figure plots various performance metrics of the edgeR package with TMM normalization as a function of the fraction of features that remain unchanged across conditions (π), and the lower (a) and upper bounds (b) of a Uniform fold change distribution. Control proportions (q_1) were obtained from rat liver tissue of the rat bodymap [15]. In contrast to what was observed with total sum approaches, the false positive rates are maintained at low levels for a larger range of parameters. Sensitivity values still remained low. High false positive rates result with higher variance and asymmetrically located (with respect to 0) fold change distributions. The results were similar across testing platforms, for median based normalization techniques like that of DESeq/Median scaling, and for the Gaussian fold change distribution. 24

5 **Reference normalization (TMM/DESeq/Median) under a Gaussian fold change distribution.** The figure plots various performance metrics of the edgeR package as a function of the fraction of features that remain unchanged across conditions (π), and the mean (μ) and standard deviation (σ) of the Gaussian fold change distribution for the same control proportions (q_1) as in figure 4. (A) (σ, π) variations at $\mu = 0$. (B) (μ, π) variations at a constant $\sigma = 1$. When the fraction of unperturbed features is large, in contrast to what was observed with total sum approaches, higher fold change distribution variances and means lead to better performance. As supplementary figure 6 shows, many of these calls had wrong signed fold changes. Higher means and variances of fold change distributions are therefore cases that lead to heavily confounded inference. The results were qualitatively similar across testing techniques. 25

6 **Confounded inference with total sum and reference normalization strategies.** For all features whose reconstructed fold changes had wrong signs when called significant, together with false negatives, we plot the sampled (first column) fold changes and deviations in the edgeR reconstructed fold changes from those of the true values after total sum (second column) and TMM (third column) normalizations. The corresponding parameter values for the simulations are shown alongside the plots. Larger deviations from the horizontal line at 0 imply higher confounding in inference. Asymmetric FCDs, which give rise to feature specific fold changes biased to be more positive or negative, can easily trick inference based on total sum based normalization approaches. TMM and other voting based strategies behave in a more robust fashion. However, when larger fraction of features (25%) varies across conditions, their performance becomes highly sensitive to the underlying FCDs. 26

7 **Scale factors obtained from pseudocounted sparse datasets are severely biased.** Description same as that in Fig. 5 in the main text except the pseudocount value has been altered to 10^{-7}. 27
8 **Simulation performance in the Diarrheal microbiomes.** Description same as that in Fig. 7 in the main text, except that the control proportions were set to those arising from the diarrheal study. 28
9 **Simulation comparisons with CLR.** Description same as that in Fig. 7 in the main text. Because logarithmic transforms are used with CLR, and lognormal assumptions are often made on these transformations, we used it along with Limma in these simulations. As is commonly done with these transformations [16], we used a pseudo count (of 1) to avoid zero multiplications and divisions. The behavior was similar if exponentiated CLR factors were input as scale factors to edgeR as well. 29
10 **Groupwise integrity in the compositional scales of the Human Microbiome Project's samples from the J. Craig Venter Institute.** To be compared with Fig. 8 in the main text. On the top-left, we plot the logged median of the positive ratios of group-averaged proportions to that of Throat chosen as the reference group. Stool samples show considerable deviation in their compositional scales from the rest of the samples. Minor variations in the relative placements were observed across centers potentially due to technical sources of variation, however the overall behavior of the Stool samples were similar across sequencing centers. Corresponding CSS scales in **supplementary 11**. 30
11 **CSS compositional scale reconstructions.** (**A**) Baylor College of Medicine Samples, and (**B**) J. Craig Venter Institute's Samples 31
12 **Groupwise integrity in the compositional scales in the Mouse microbiomes.** The numbers on the labels mark the day of the time series observation. 32
13 **Groupwise integrity in the compositional scales in Lung Microbiomes.** We have not shown the Scran specific plot as the technique had particular difficulties with the sparsity level in this dataset. 33
14 **Groupwise integrity in the compositional scales in the Diarrheal Microbiomes.** Both the sample type, and the country of origin are shown. We did not observe significant differences in the compositional scales assigned to the various groups, across all techniques. 34
15 **Importance of compositional correction in common bulk RNAseq studies.** (**A**) Application of scaling techniques to the rat body map data across tissues. Median positive ratio: median of the positive ratios of group-averaged proportions to that of Adrenal chosen as the reference. Subsequent figures in the top row indicate higher sparsity levels in the heart, muscle and liver samples, although at sequencing depths that are comparable/slightly higher to those from other tissue groups. (**B**) Reconstructed scales from several normalization techniques. If one were to perform a differential expression analysis between Testes and Heart, the fold changes are roughly 4X (ratio of medians) inflated as predicted by Scran/Wrench, which can lead to high false positive rates especially if most features are not changed across the two tissues.Notice the similarity in scales for closely related tissues, across techniques; for these tissues, the influence of compositional bias in the related differential abundance tests will be low. 35
16 **Interplay of compositional bias and observation heterogeneity.** Compositional bias overlaying the asynchronous nature of samples (with respect to the underlying biological dynamics) chosen for cross-sectional observations can induce zero-inflation in metagenomic and single cell sequencing. The figure demonstrates this behavior with a few candidate genes/taxa. The problem will be severe in real-life systems given the large number of genes and microbes teeming in the chosen ecosystems of interest. . . 36
17 **Construction of the regression equations by solving a bipartite matching problem.** Under the assumption of feature-specific hurdle-log-normal feature distributions, the expectation of "adjusted" DESeq-style factors ($\tilde{h}_{ij}$ in text) estimated for every feature i in every data sample j is log-linear in the logged-compositional scales. However, $\tilde{h}_{ij}$s are correlated across j, as they share ratios; this means, we first need to solve a problem of finding a feature i for every sample j such that the resulting set of equations are constructed from roughly independent data. This is achieved by solving an unweighted bipartite matching problem, where every feature i is matched with a sample j. In the graph, an edge occurs between Λ_j and feature node i whenever i has a positive count in sample j. The dark edges (green-lit matrix cells) represent the matched features. If needed, each such edge can be weighted, for example, by inverse binomial variance of feature i in sample j. Notice that if $degree(\Lambda_j) \geq n$ for all samples j, we can randomly match a unique expressed i with each sample j as a solution. In the metagenomics datasets we consider here, $degree(\Lambda_j) \ll n$. 37
18 **Bland-Altman plot for Tara correlative analysis in Table 3 of main text .** The y-axis plots the differences between the reconstructed scales and the experimentally measured values. The x-axis plots the average of the two. 38
19 **Bland-Altman plot for UMI single cell RNAseq correlative analysis in Table 3 of main text .** The y-axis plots the differences between the reconstructed scales and the experimentally measured values. The x-axis plots the average of the two. 39

20 **Bland-Altman plot for Rat Bodymap correlative analysis in Table 3 of main text .** The y-axis plots the differences between the reconstructed scales and the experimentally measured values. The x-axis plots the average of the two. 40
21 **Simulation performance in a balanced design.** We plot the performance metrics as a function of sample size and fraction of features f that are perturbed in cases. Sample depth fixed to 10K reads on average per sample. Legend: Red, Wrench; Black: TMM. 41
22 **Simulation performance in an unbalanced design.** We plot the performance as a function of sample size and fraction of features f that are perturbed in cases. The total number of case samples were fixed to 20, and the number of control samples were varied to simulate unbalanced designs. So in the plot, a sample size of 20 corresponds to a sample size of 20 for the case sample, and therefore reflects a balanced design. The rest represent unbalanced designs. Sample depth fixed to 10K reads on average per sample. Legend: Red, Wrench; Black: TMM. 42
23 **Benchmarking analysis of the Argyropolous et al., miRNA dataset for deviatioin from expected fold changes in the clustered symmetric DE without global changes in expression ratiometric A versus B.** Same as Fig. 7 in [14]. The shown numbers measure deviation of the reconstructed fold changes from the true expected fold changes by experimental design, for the pipeline. Lower is better. Refer [14], Fig. 7 for details on experimental design. 43
24 **Spike-in proportions show trends with sample depth.** The y-axis plots the differences between the logged spike-in count and sample depth, and the x-axis represents sample-depth factors (upto arbitrary scaling). 44

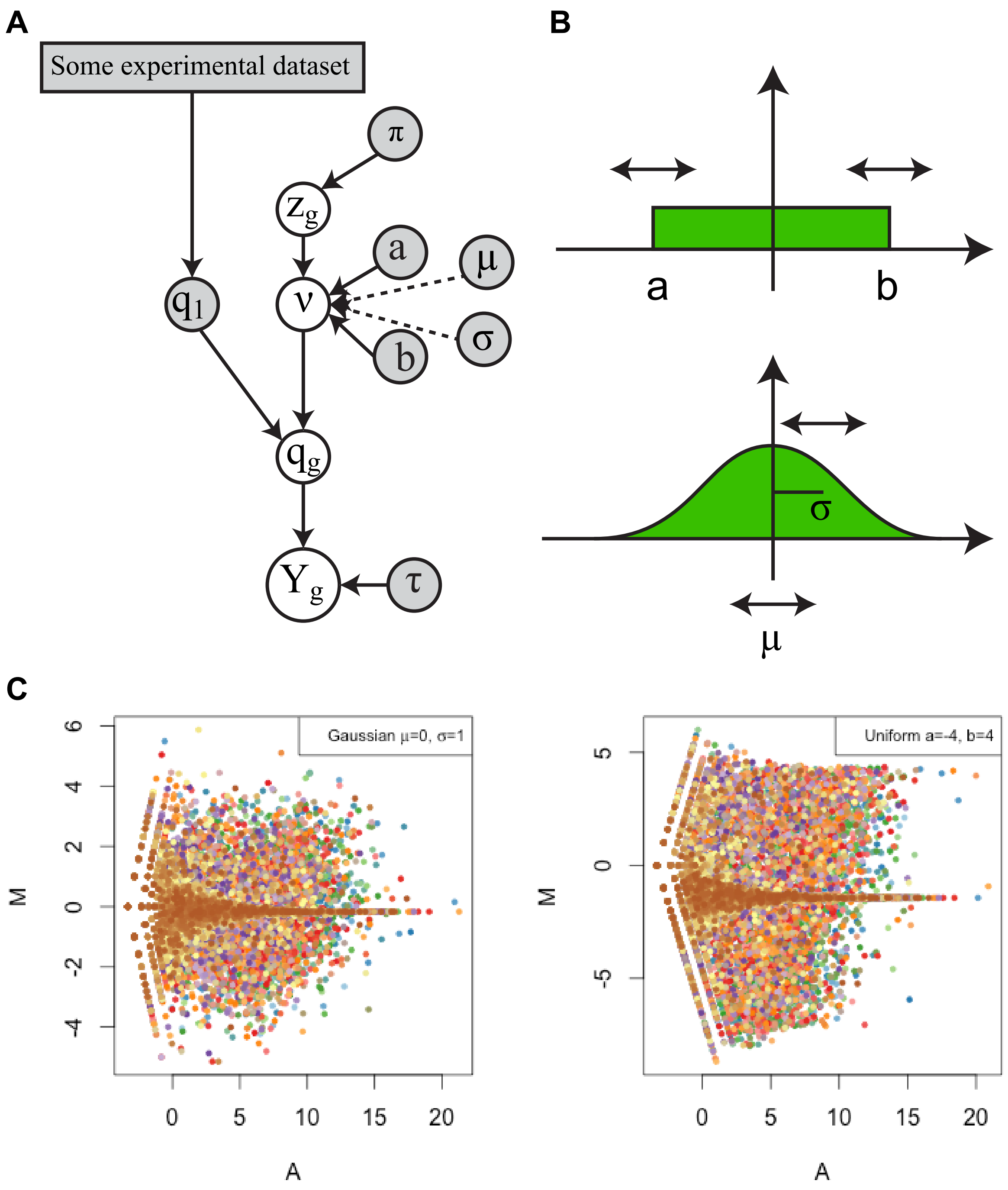

Figure 1: Simulation strategy for evaluating current normalization and differential expression analysis toolkits for compositional correction. (A) Simulation set up. q_1, q_g represent the control and case proportion vector of all the features. q_1 is obtained from a given experimental dataset. π represents the fraction of features that do not change across conditions. Z_g $Bernoulli(\pi)$ represents the set of indicator variables that denote if a feature is not differentially expressed. Conditioned on Z_g, the logged vector of fold changes $\log \nu$ is sampled from a two-parameter fold change distribution, with ν_{gi} set to 1 whenever Z_{gi} is 1. Here i indexes the individual entries of the vector. The sampled fold changes and control proportions are normalized to yield the case proportions. A multinomial draw for a fixed sample depth τ (20M reads) then yields the desired simulated sequencing output. The two fold change distributions, $Unif(a,b)$ and a $N(\mu,\sigma^2)$, considered in our study are shown in (B). Example simulations when 75% (i.e., $\pi = 0.75$) of the features are fixed across conditions, with the rest perturbed according to log fold changes sampled from $Normal(0,1)$ and $Unif(-4,4)$ fold change distributions respectively are shown in (C).

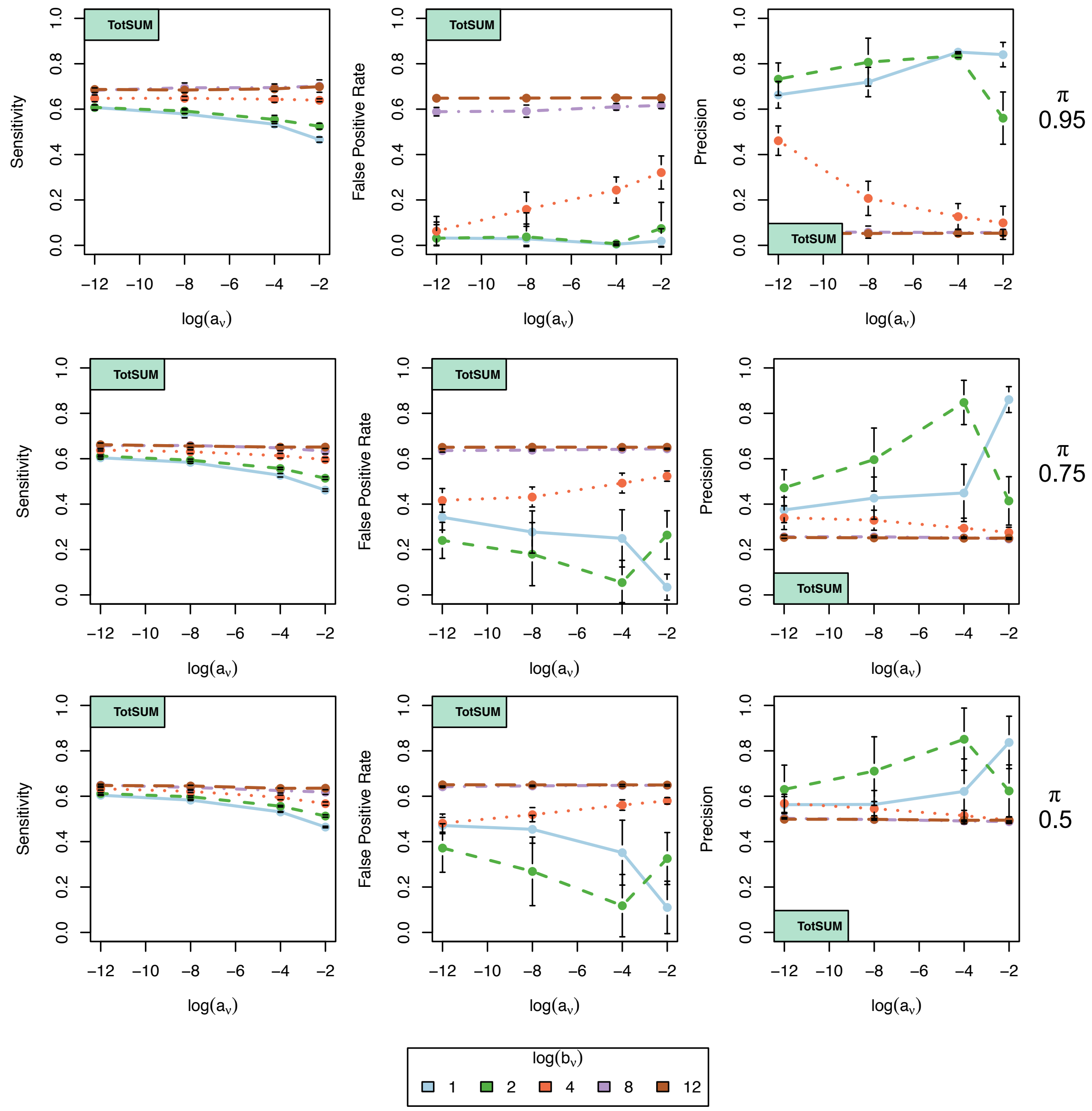

Figure 2: Total sum based normalization, like RPKM/Rarefication, under a Uniform fold change distribution. The figure plots various performance metrics of the edgeR package as a function of the fraction of features that remain unchanged across conditions (π), and the lower (a) and upper bounds (b) of a Uniform fold change distribution. Control proportions (q_1) were obtained from rat liver tissue of the rat bodymap [15]. Extremely high false positive rates result with higher variance and asymmetrically located fold change distributions (i.e., with positive or negative means) due to compositionality induced confounding. The results were similar across testing platforms, and for the Gaussian fold change distribution(fig. 3).

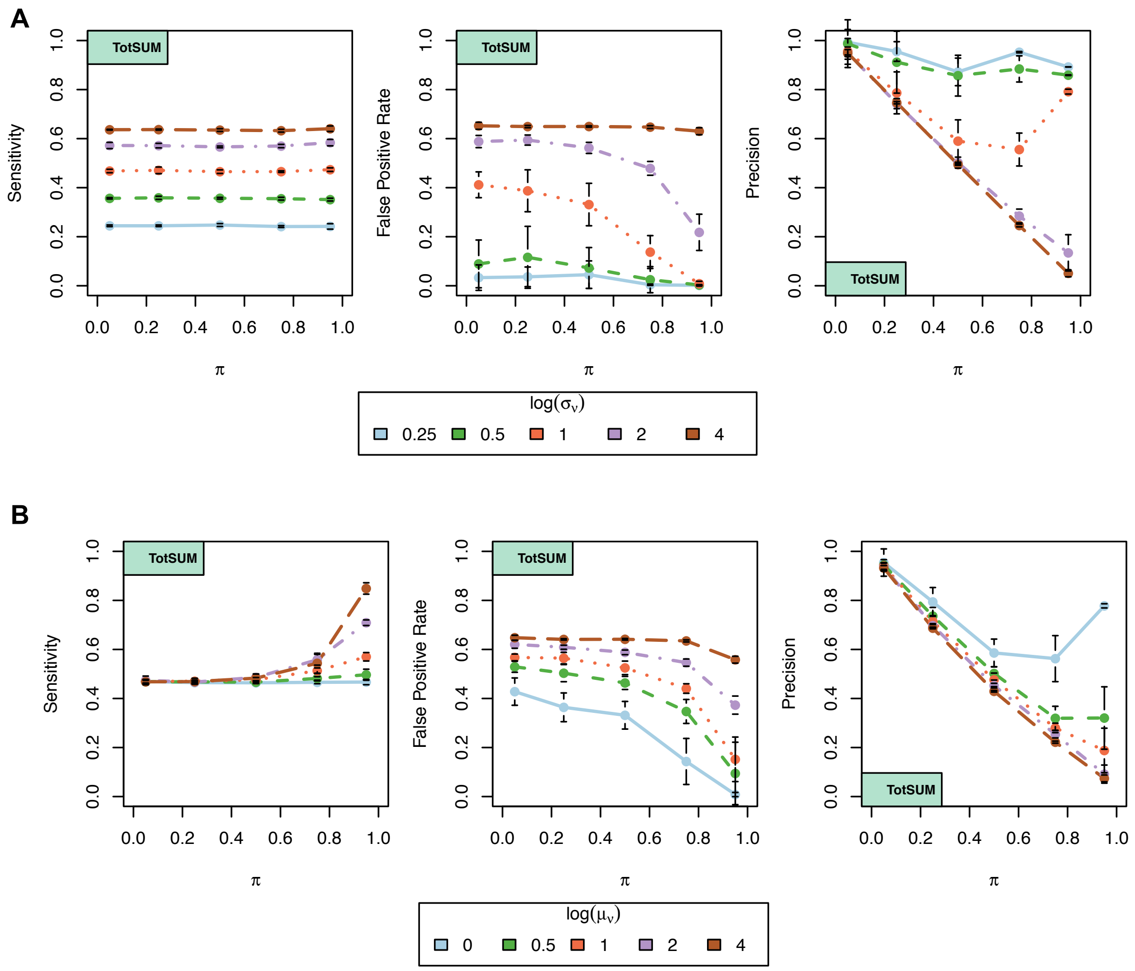

Figure 3: Total sum based normalization, like RPKM/Rarefication, under a Gaussian fold change distribution. The figure plots various performance metrics of the edgeR package as a function of the fraction of features that remain unchanged across conditions (π), and the mean (μ) and standard deviation (σ) of the Gaussian fold change distribution for the same control proportions (q_1) as in figure 5. (A) (σ, π) variations at $\mu = 0$. (B) (μ, π) variations at $\sigma = 1$. It would appear that higher fold change distribution variances and means lead to better performance, but these are also associated with higher false positive rates and as figure 6shows, large fraction of these calls had wrong signed fold changes. Higher means and variances of fold change distributions are therefore cases that lead to heavily confounded inference. The results were similar across testing techniques.

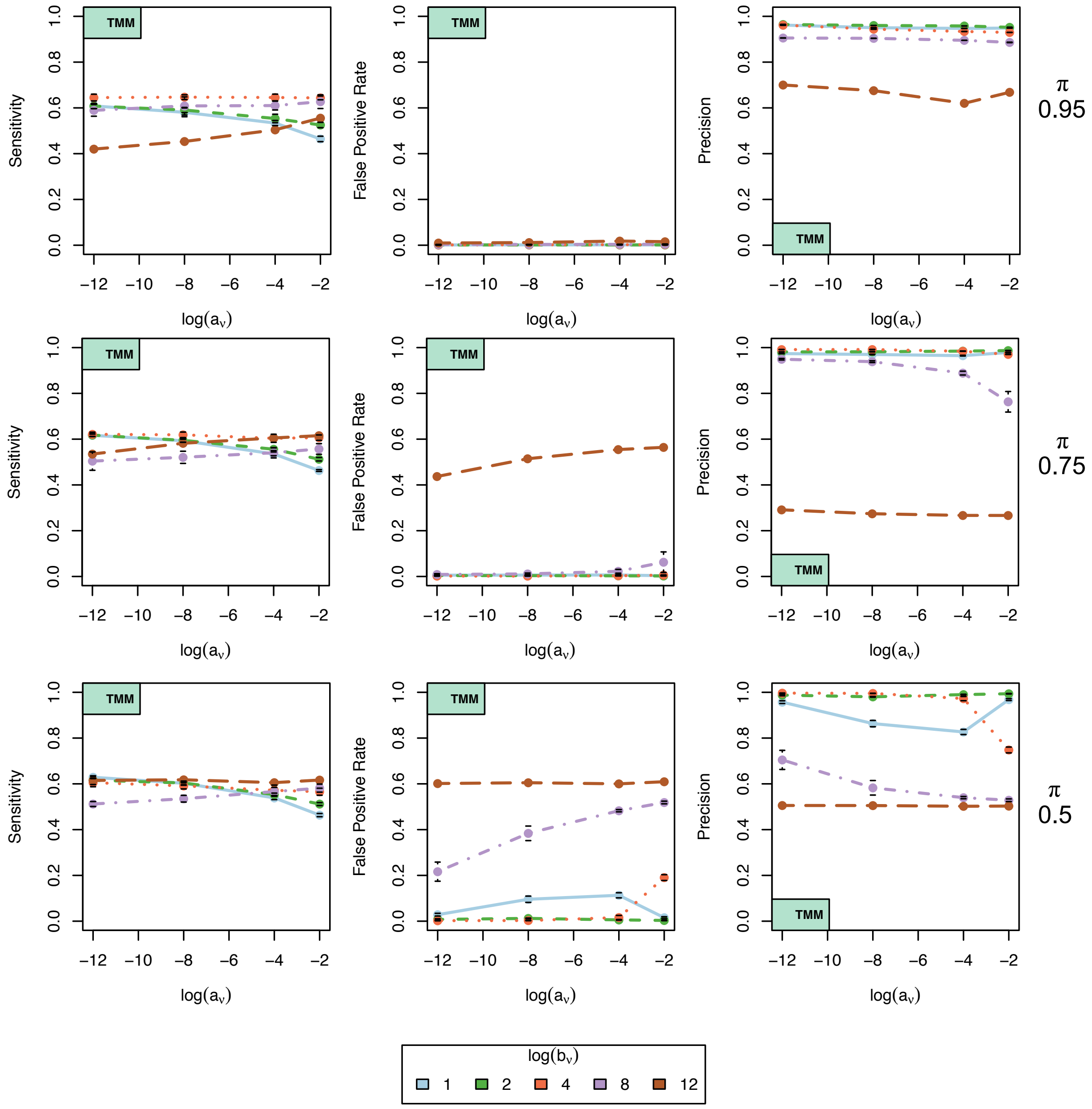

Figure 4: Reference normalization (TMM/DESeq/Median) under a Uniform fold change distribution. The figure plots various performance metrics of the edgeR package with TMM normalization as a function of the fraction of features that remain unchanged across conditions (π), and the lower (a) and upper bounds (b) of a Uniform fold change distribution. Control proportions (q_1) were obtained from rat liver tissue of the rat bodymap [15]. In contrast to what was observed with total sum approaches, the false positive rates are maintained at low levels for a larger range of parameters. Sensitivity values still remained low. High false positive rates result with higher variance and asymmetrically located (with respect to 0) fold change distributions. The results were similar across testing platforms, for median based normalization techniques like that of DESeq/Median scaling, and for the Gaussian fold change distribution.

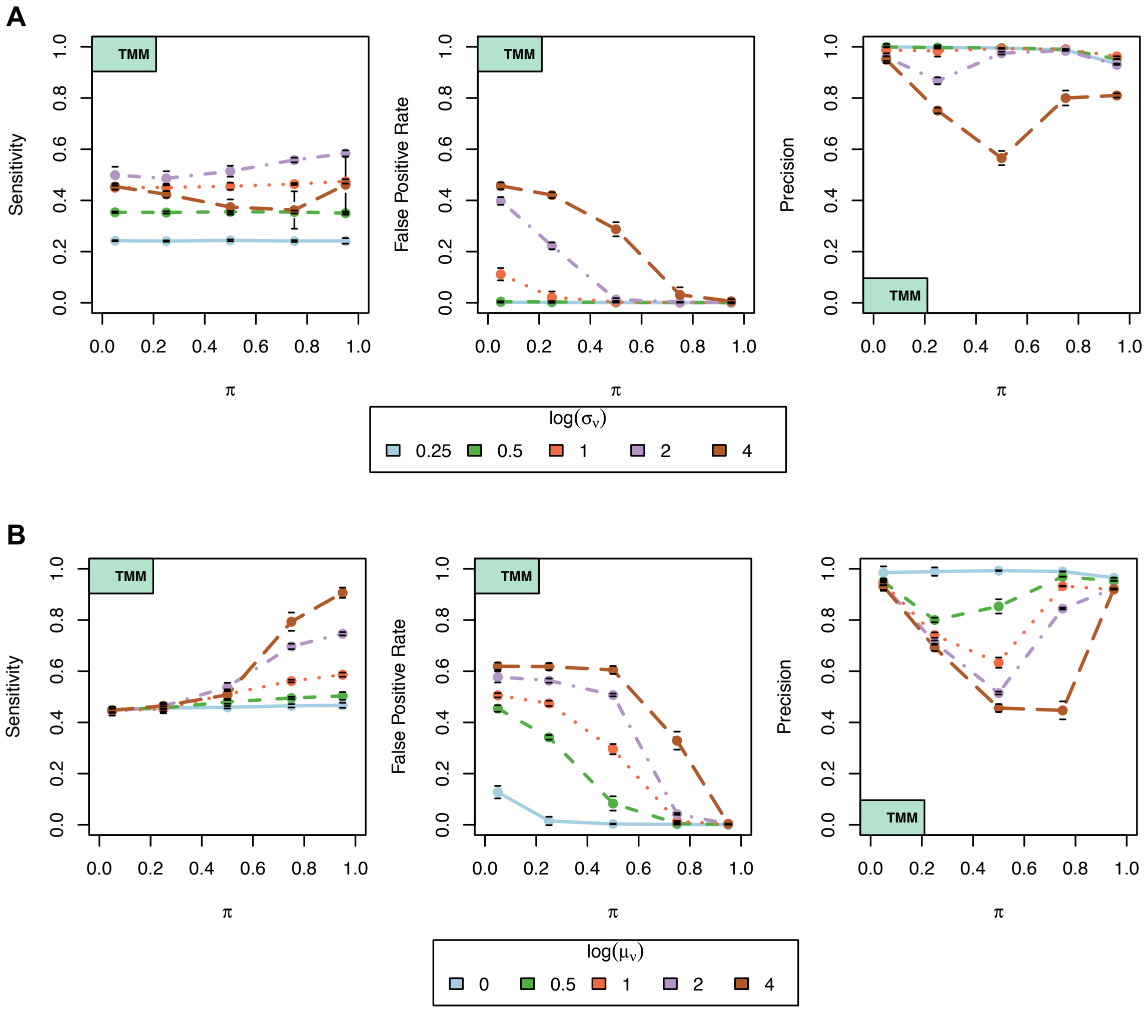

Figure 5: Reference normalization (TMM/DESeq/Median) under a Gaussian fold change distribution. The figure plots various performance metrics of the edgeR package as a function of the fraction of features that remain unchanged across conditions (π), and the mean (μ) and standard deviation (σ) of the Gaussian fold change distribution for the same control proportions (q_1) as in figure 4. (A) (σ, π) variations at $\mu = 0$. (B) (μ, π) variations at a constant $\sigma = 1$. When the fraction of unperturbed features is large, in contrast to what was observed with total sum approaches, higher fold change distribution variances and means lead to better performance. As supplementary figure 6 shows, many of these calls had wrong signed fold changes. Higher means and variances of fold change distributions are therefore cases that lead to heavily confounded inference. The results were qualitatively similar across testing techniques.

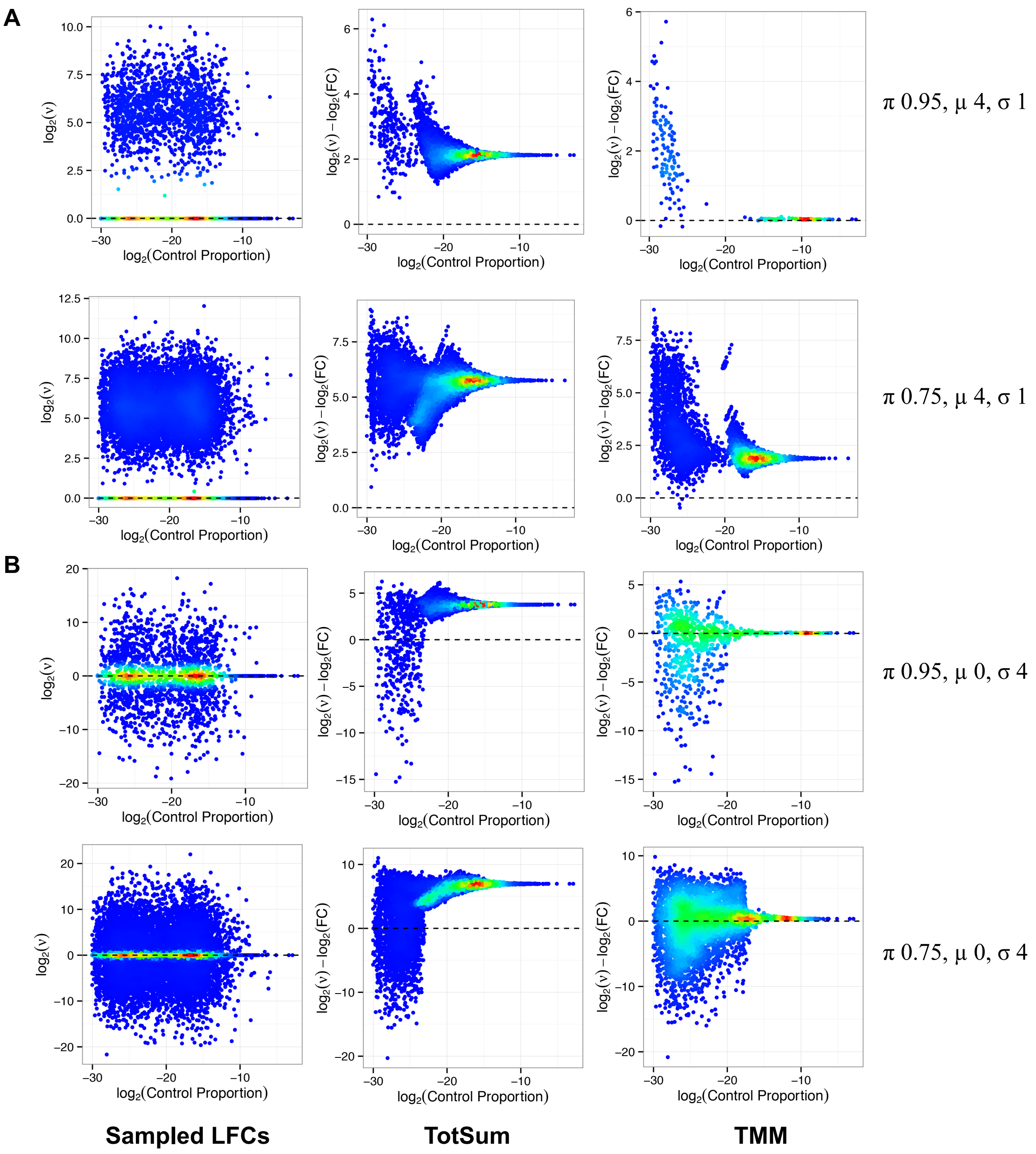

Figure 6: Confounded inference with total sum and reference normalization strategies. For all features whose reconstructed fold changes had wrong signs when called significant, together with false negatives, we plot the sampled (first column) fold changes and deviations in the edgeR reconstructed fold changes from those of the true values after total sum (second column) and TMM (third column) normalizations. The corresponding parameter values for the simulations are shown alongside the plots. Larger deviations from the horizontal line at 0 imply higher confounding in inference. Asymmetric FCDs, which give rise to feature specific fold changes biased to be more positive or negative, can easily trick inference based on total sum based normalization approaches. TMM and other voting based strategies behave in a more robust fashion. However, when larger fraction of features (25%) varies across conditions, their performance becomes highly sensitive to the underlying FCDs.

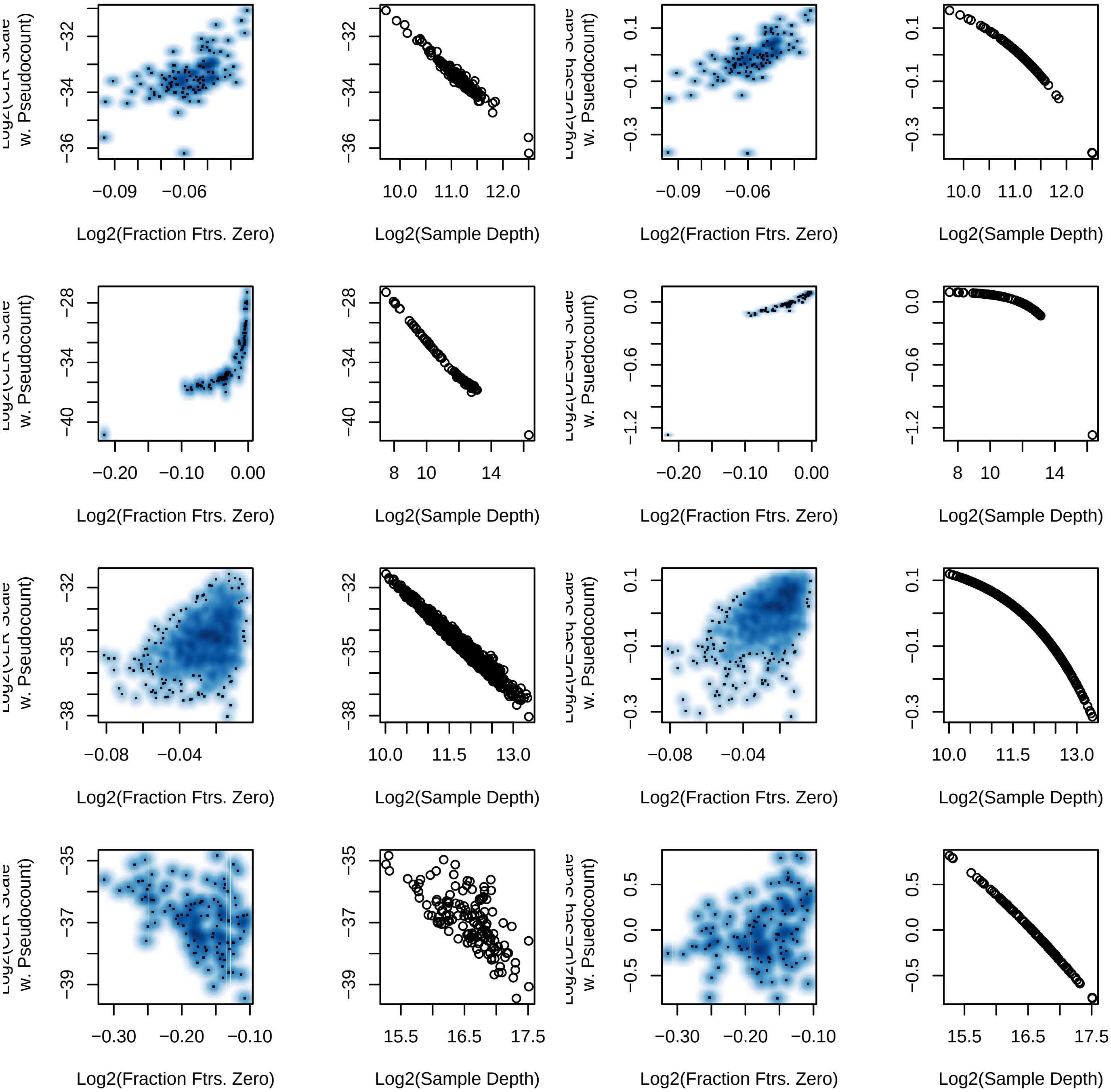

Figure 7: Scale factors obtained from pseudocounted sparse datasets are severely biased. Description same as that in Fig. 5 in the main text except the pseudocount value has been altered to 10^{-7}.

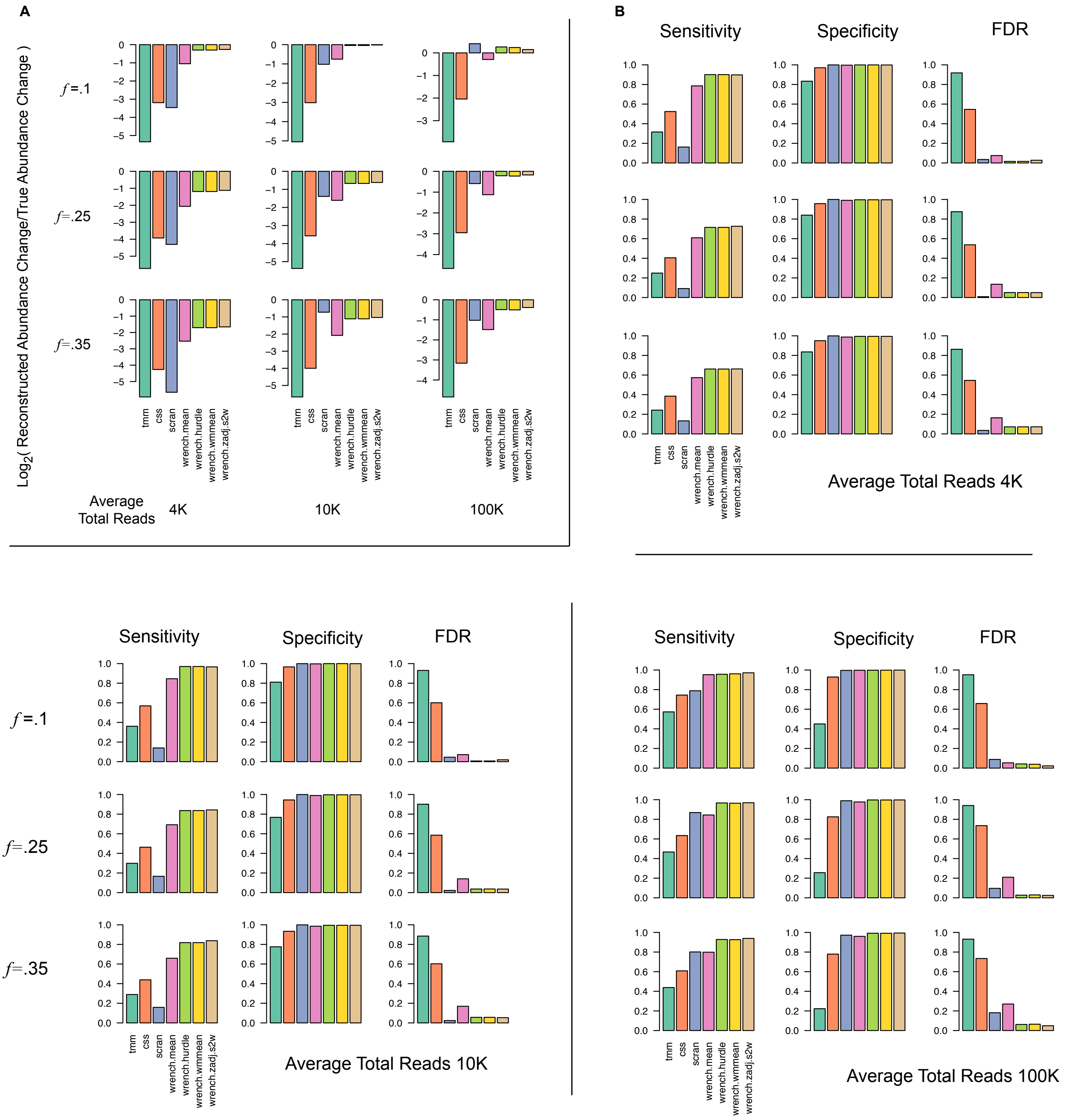

Figure 8: Simulation performance in the Diarrheal microbiomes. Description same as that in Fig. 7 in the main text, except that the control proportions were set to those arising from the diarrheal study.

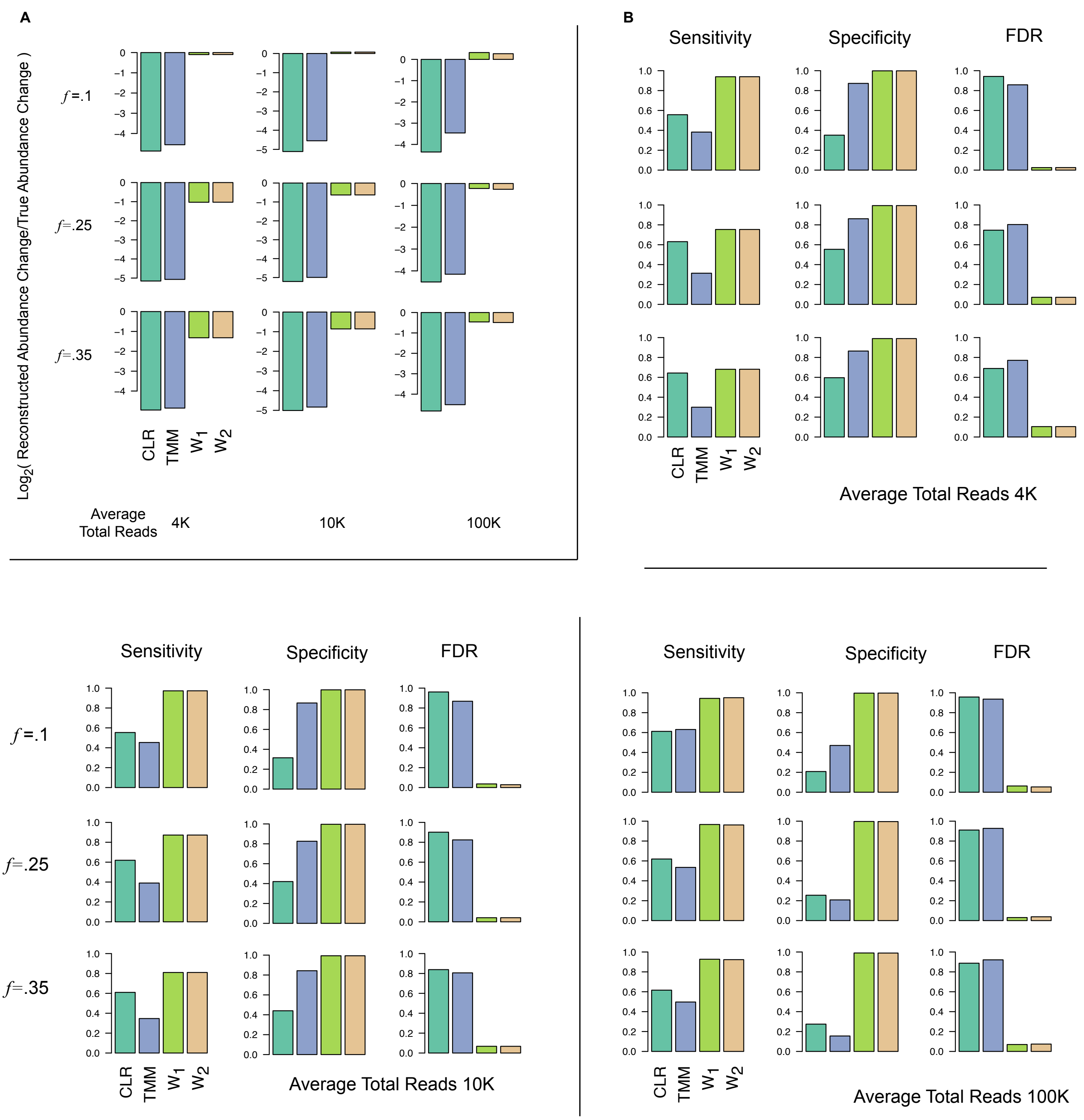

Figure 9: Simulation comparisons with CLR. Description same as that in Fig. 7 in the main text. Because logarithmic transforms are used with CLR, and lognormal assumptions are often made on these transformations, we used it along with Limma in these simulations. As is commonly done with these transformations [16], we used a pseudo count (of 1) to avoid zero multiplications and divisions. The behavior was similar if exponentiated CLR factors were input as scale factors to edgeR as well.

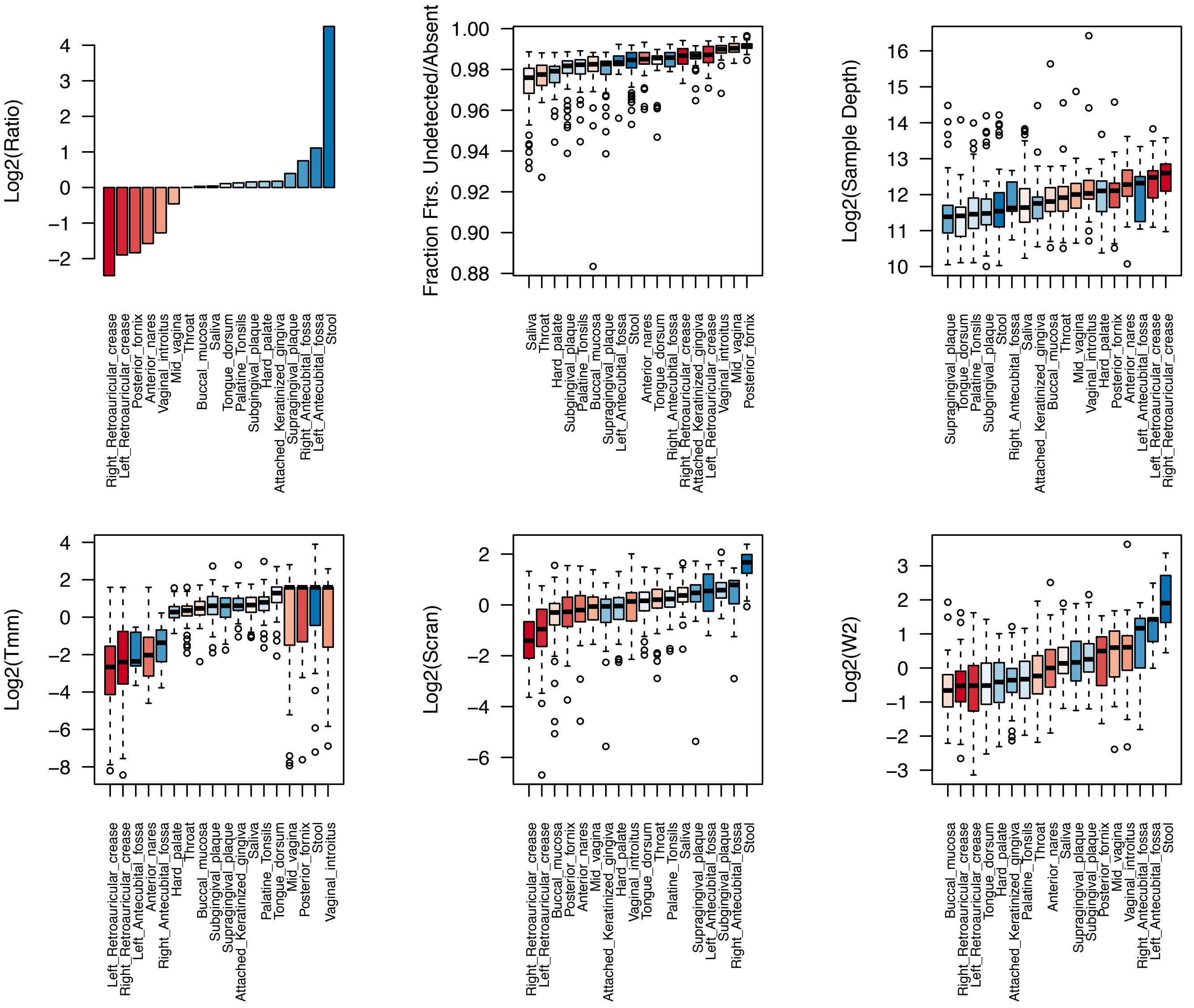

Figure 10: Groupwise integrity in the compositional scales of the Human Microbiome Project's samples from the J. Craig Venter Institute. To be compared with Fig. 8 in the main text. On the top-left, we plot the logged median of the positive ratios of group-averaged proportions to that of Throat chosen as the reference group. Stool samples show considerable deviation in their compositional scales from the rest of the samples. Minor variations in the relative placements were observed across centers potentially due to technical sources of variation, however the overall behavior of the Stool samples were similar across sequencing centers. Corresponding CSS scales in **supplementary 11**.

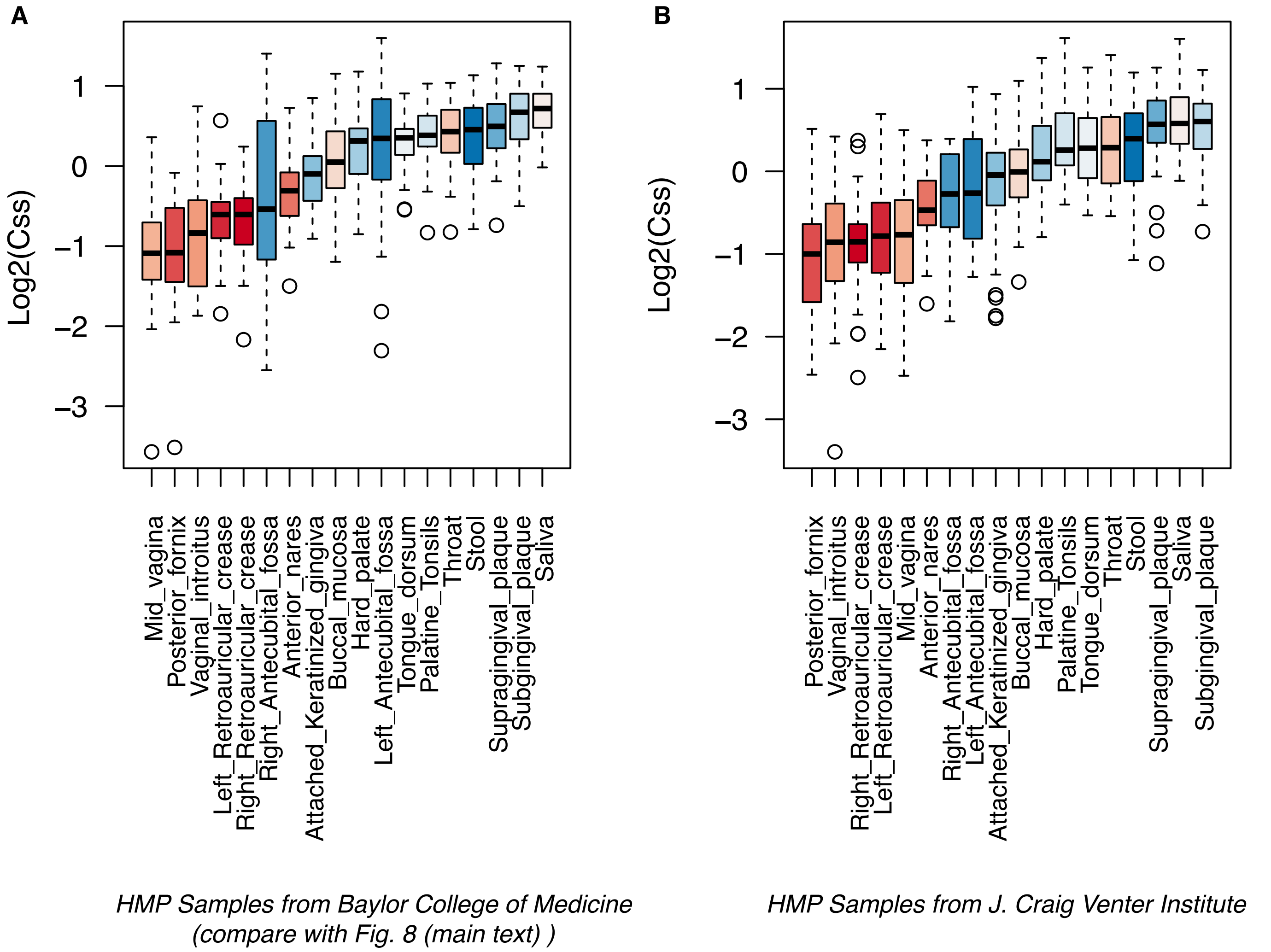

Figure 11: CSS compositional scale reconstructions. (**A**) Baylor College of Medicine Samples, and (**B**) J. Craig Venter Institute's Samples

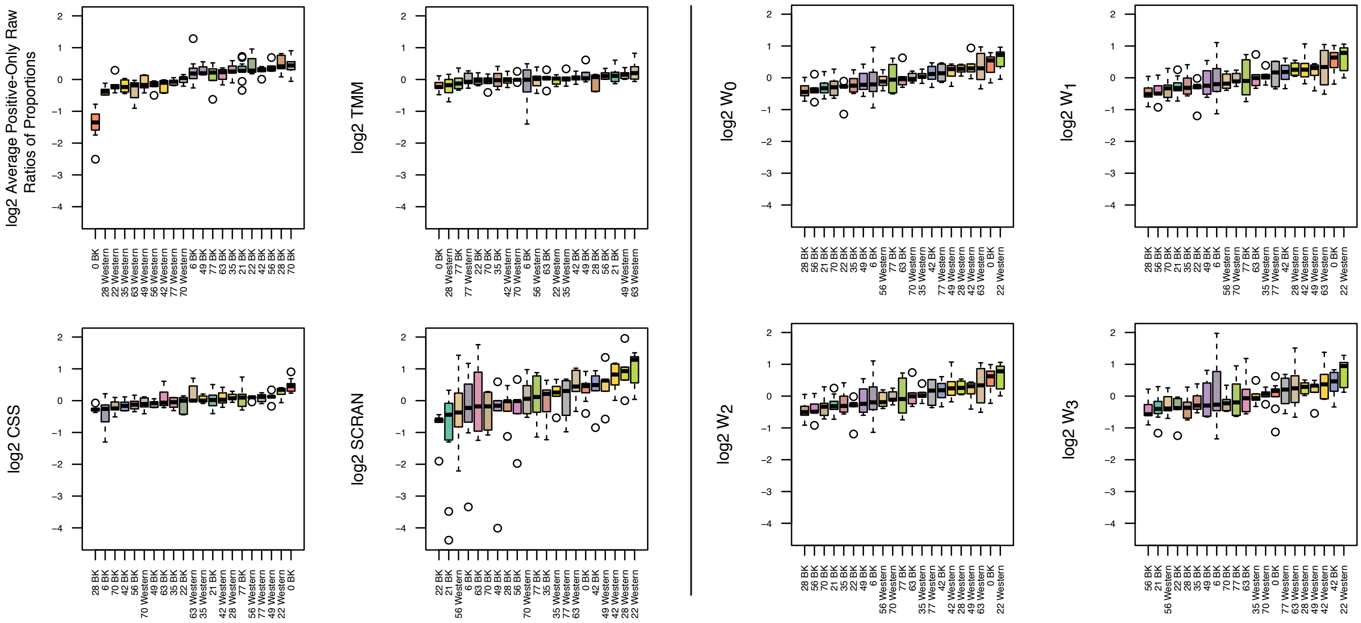

Figure 12: Groupwise integrity in the compositional scales in the Mouse microbiomes. The numbers on the labels mark the day of the time series observation.

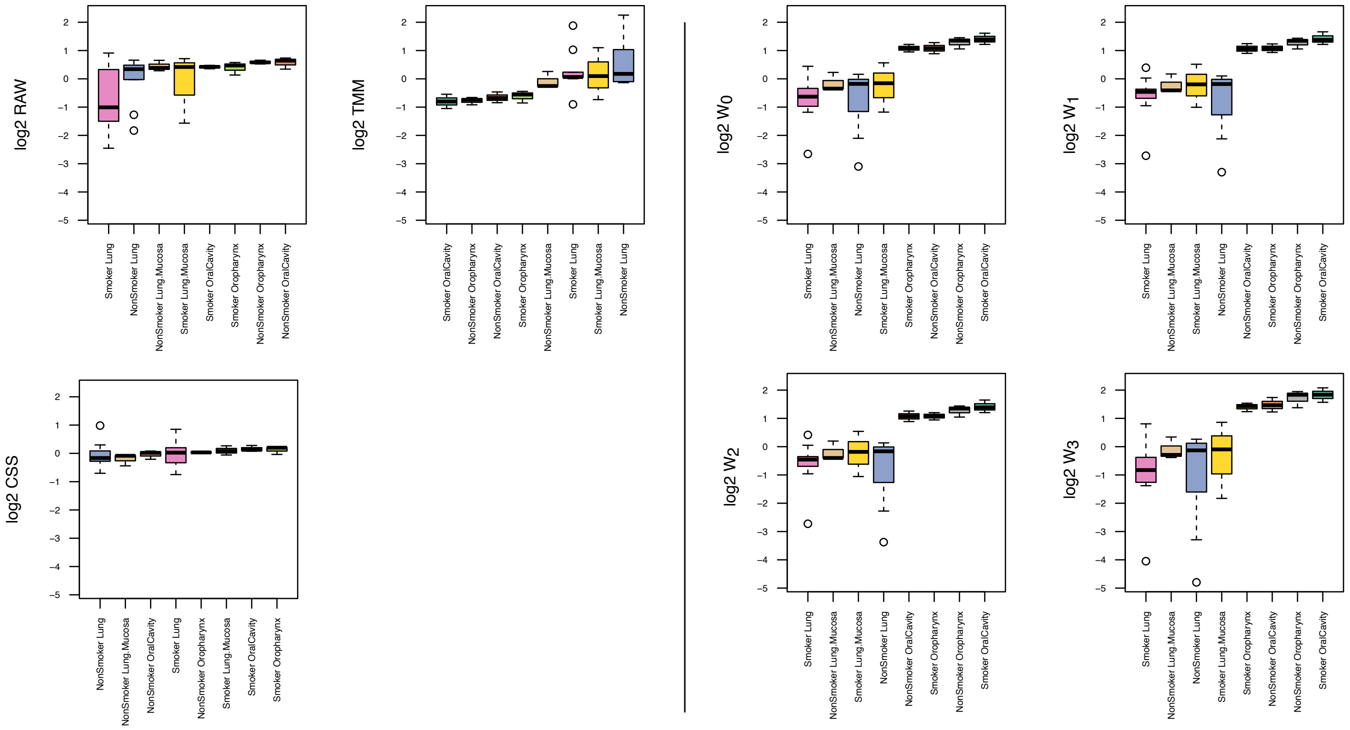

Figure 13: Groupwise integrity in the compositional scales in Lung Microbiomes. We have not shown the Scran specific plot as the technique had particular difficulties with the sparsity level in this dataset.

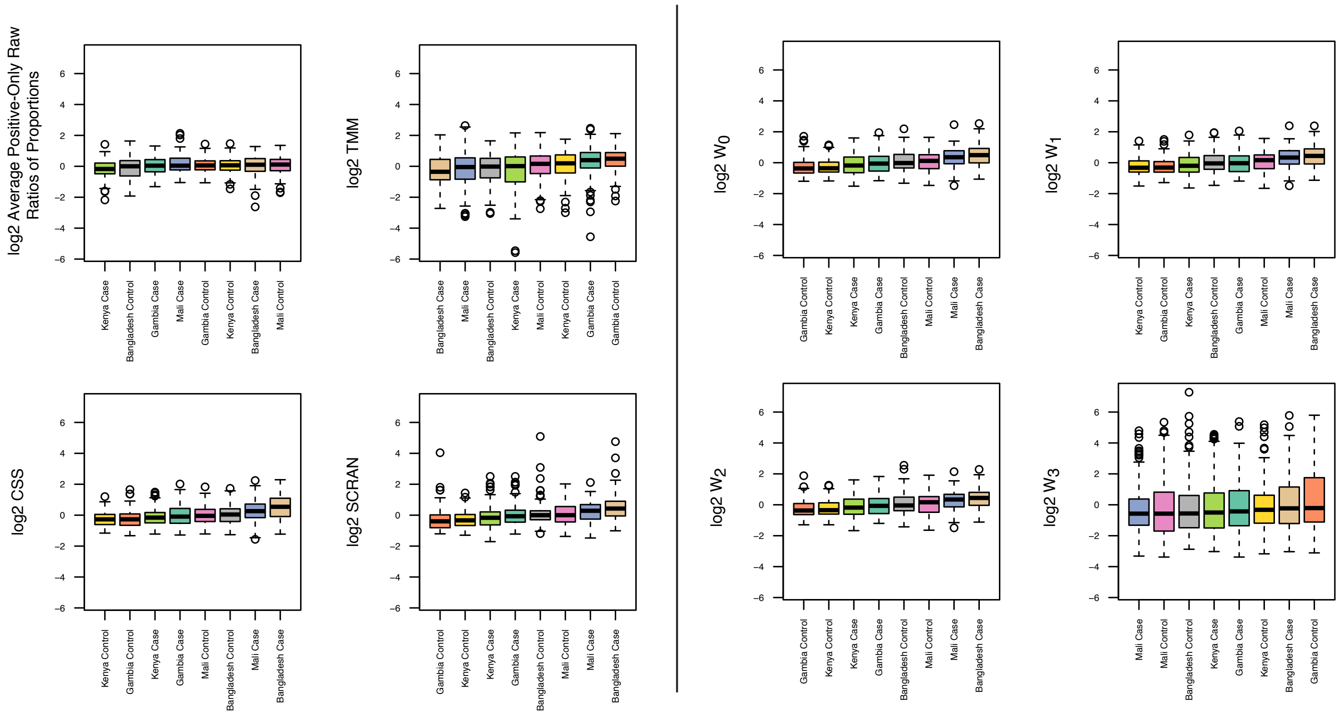

Figure 14: Groupwise integrity in the compositional scales in the Diarrheal Microbiomes. Both the sample type, and the country of origin are shown. We did not observe significant differences in the compositional scales assigned to the various groups, across all techniques.

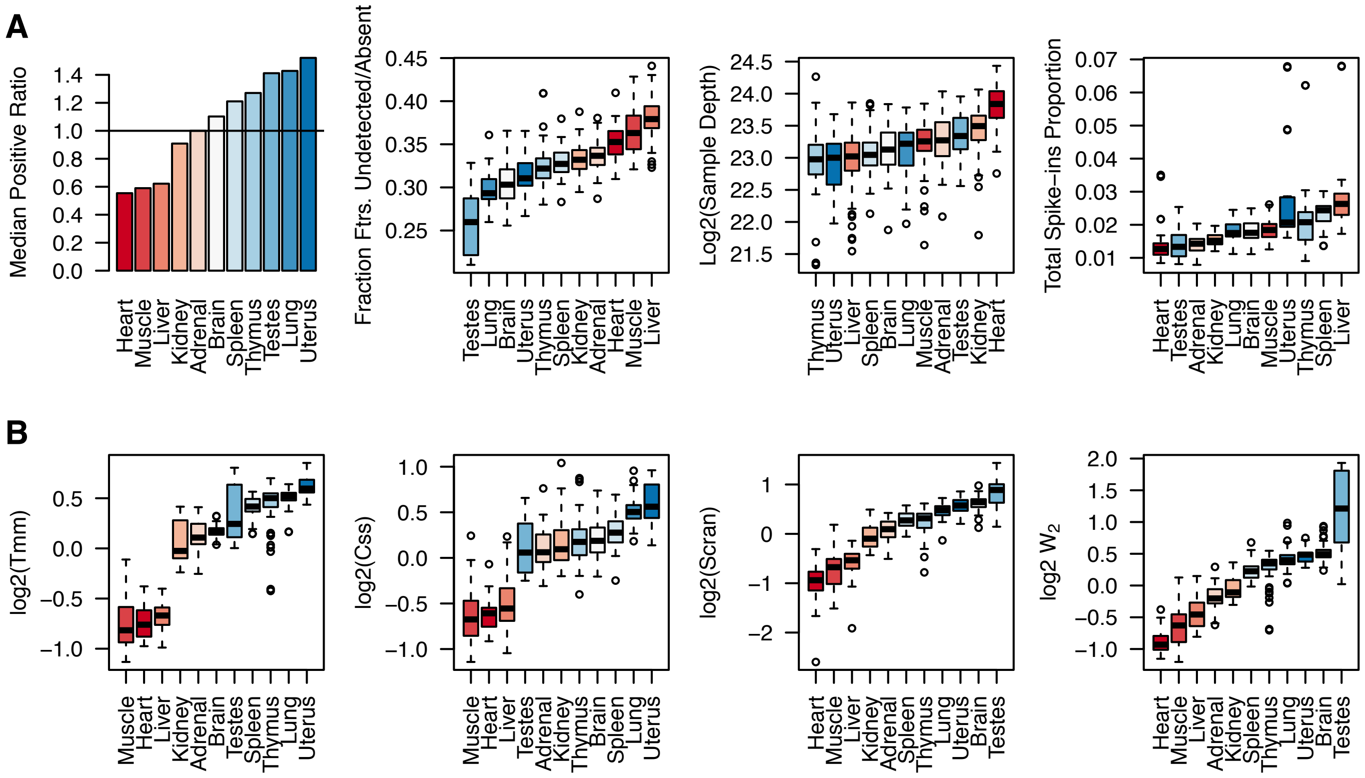

Figure 15: Importance of compositional correction in common bulk RNAseq studies. (**A**) Application of scaling techniques to the rat body map data across tissues. Median positive ratio: median of the positive ratios of group-averaged proportions to that of Adrenal chosen as the reference. Subsequent figures in the top row indicate higher sparsity levels in the heart, muscle and liver samples, although at sequencing depths that are comparable/slightly higher to those from other tissue groups. (**B**) Reconstructed scales from several normalization techniques. If one were to perform a differential expression analysis between Testes and Heart, the fold changes are roughly 4X (ratio of medians) inflated as predicted by Scran/Wrench, which can lead to high false positive rates especially if most features are not changed across the two tissues.Notice the similarity in scales for closely related tissues, across techniques; for these tissues, the influence of compositional bias in the related differential abundance tests will be low.

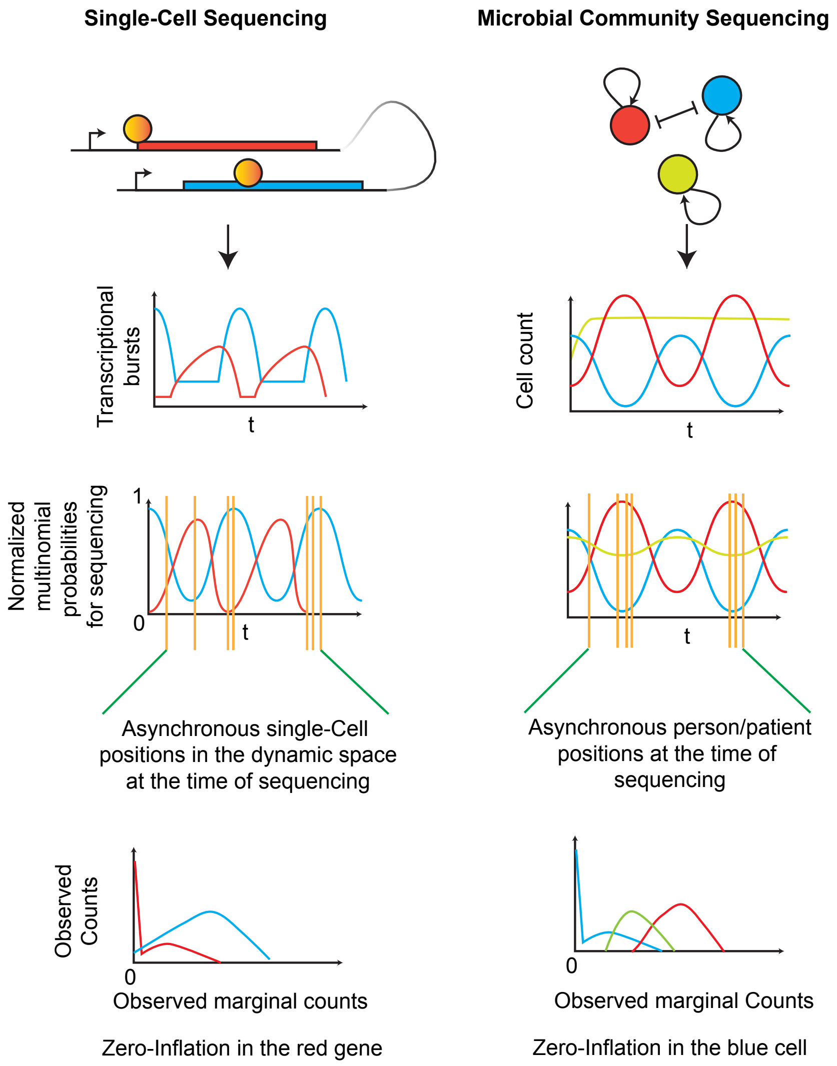

Figure 16: Interplay of compositional bias and observation heterogeneity. Compositional bias overlaying the asynchronous nature of samples (with respect to the underlying biological dynamics) chosen for cross-sectional observations can induce zero-inflation in metagenomic and single cell sequencing. The figure demonstrates this behavior with a few candidate genes/taxa. The problem will be severe in real-life systems given the large number of genes and microbes teeming in the chosen ecosystems of interest.

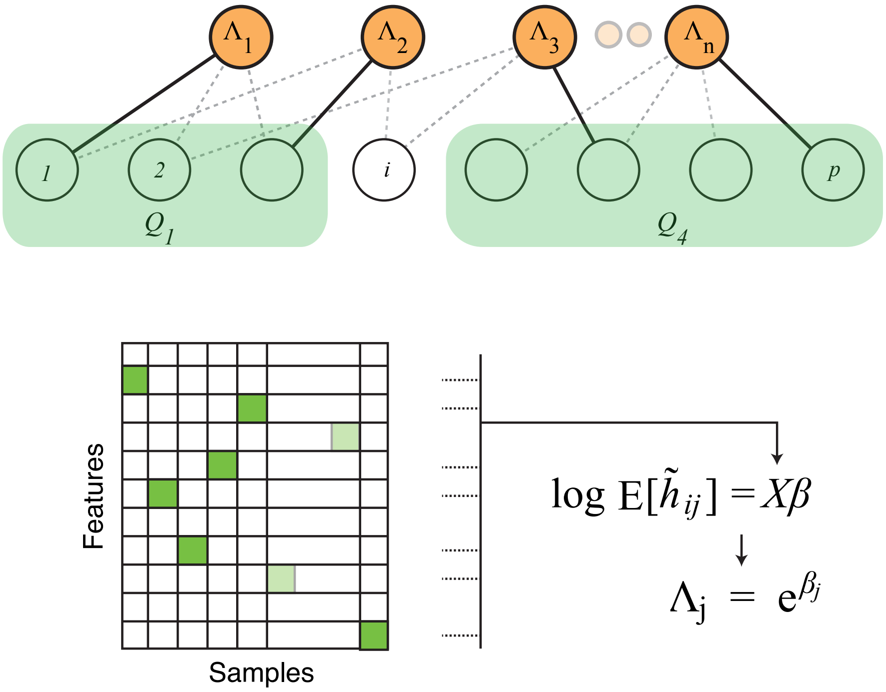

Figure 17: Construction of the regression equations by solving a bipartite matching problem. Under the assumption of feature-specific hurdle-log-normal feature distributions, the expectation of "adjusted" DESeq-style factors ($\tilde{h}_{ij}$ in text) estimated for every feature i in every data sample j is log-linear in the logged-compositional scales. However, $\tilde{h}_{ij}$s are correlated across j, as they share ratios; this means, we first need to solve a problem of finding a feature i for every sample j such that the resulting set of equations are constructed from roughly independent data. This is achieved by solving an unweighted bipartite matching problem, where every feature i is matched with a sample j. In the graph, an edge occurs between Λ_j and feature node i whenever i has a positive count in sample j. The dark edges (green-lit matrix cells) represent the matched features. If needed, each such edge can be weighted, for example, by inverse binomial variance of feature i in sample j. Notice that if $degree(\Lambda_j) \geq n$ for all samples j, we can randomly match a unique expressed i with each sample j as a solution. In the metagenomics datasets we consider here, $degree(\Lambda_j) \ll n$.

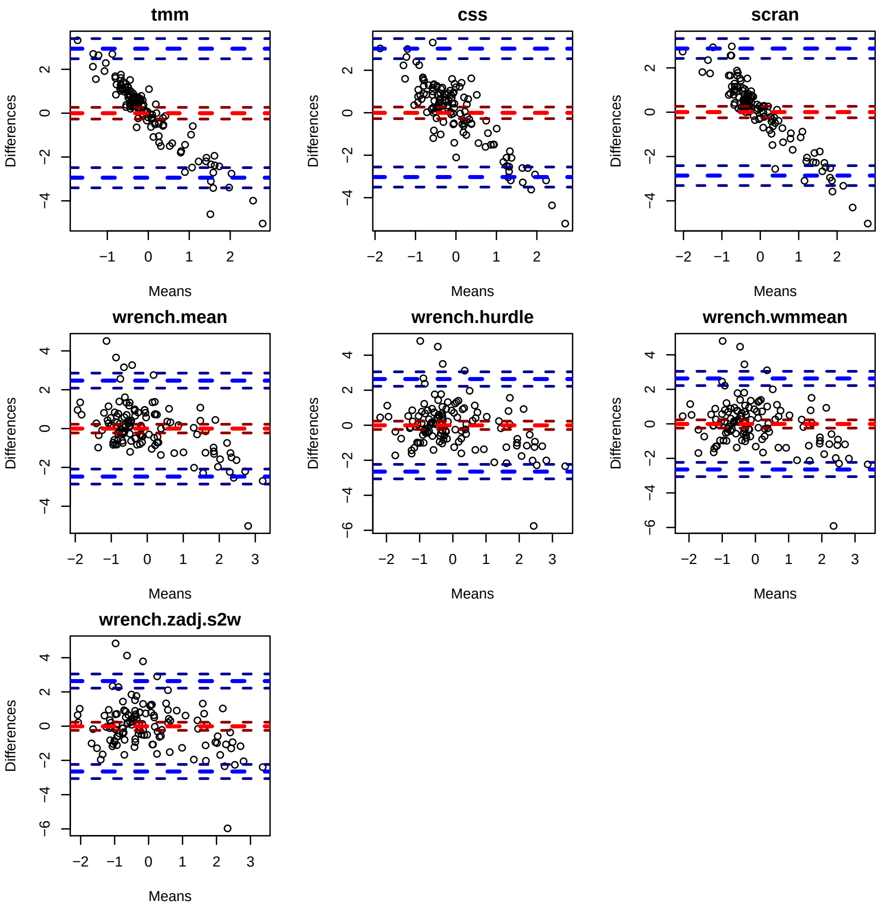

Figure 18: Bland-Altman plot for Tara correlative analysis in Table 3 of main text . The y-axis plots the differences between the reconstructed scales and the experimentally measured values. The x-axis plots the average of the two.

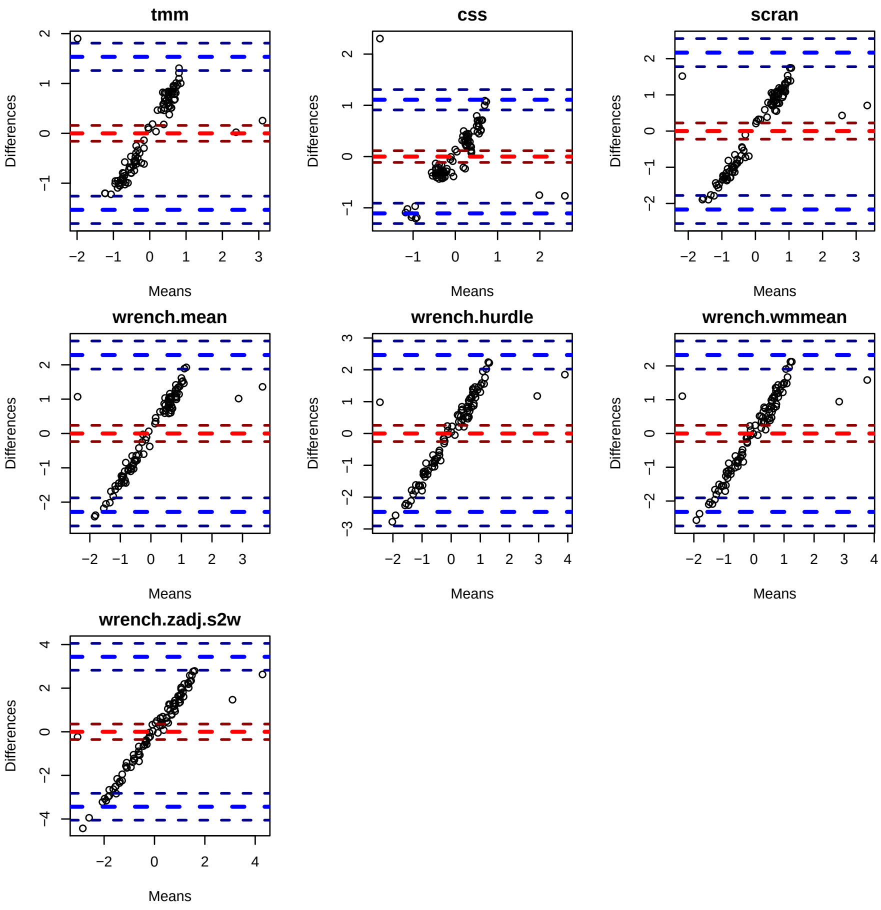

Figure 19: Bland-Altman plot for UMI single cell RNAseq correlative analysis in Table 3 of main text . The y-axis plots the differences between the reconstructed scales and the experimentally measured values. The x-axis plots the average of the two.

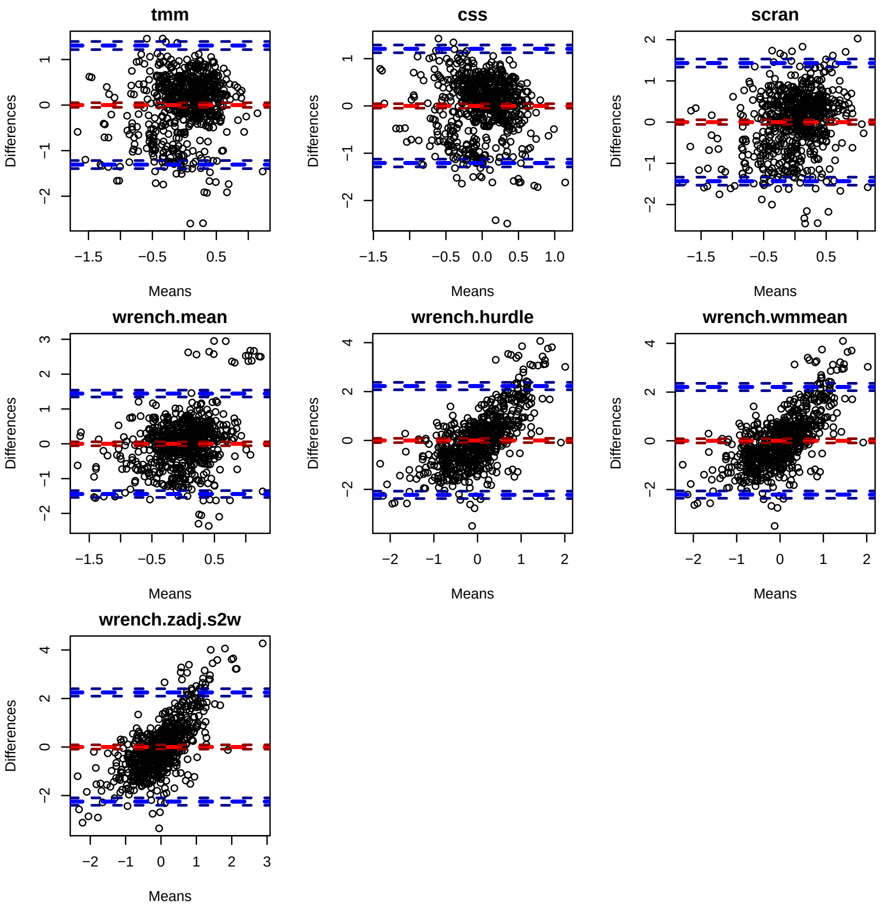

Figure 20: Bland-Altman plot for Rat Bodymap correlative analysis in Table 3 of main text . The y-axis plots the differences between the reconstructed scales and the experimentally measured values. The x-axis plots the average of the two.

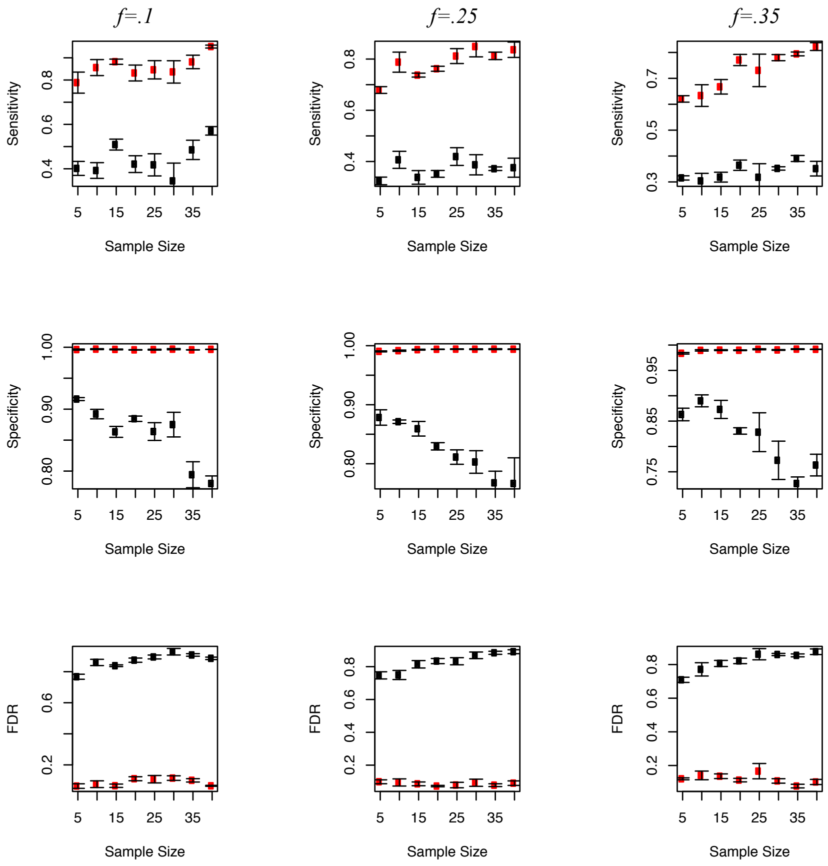

Figure 21: Simulation performance in a balanced design. We plot the performance metrics as a function of sample size and fraction of features f that are perturbed in cases. Sample depth fixed to 10K reads on average per sample. Legend: Red, Wrench; Black: TMM.

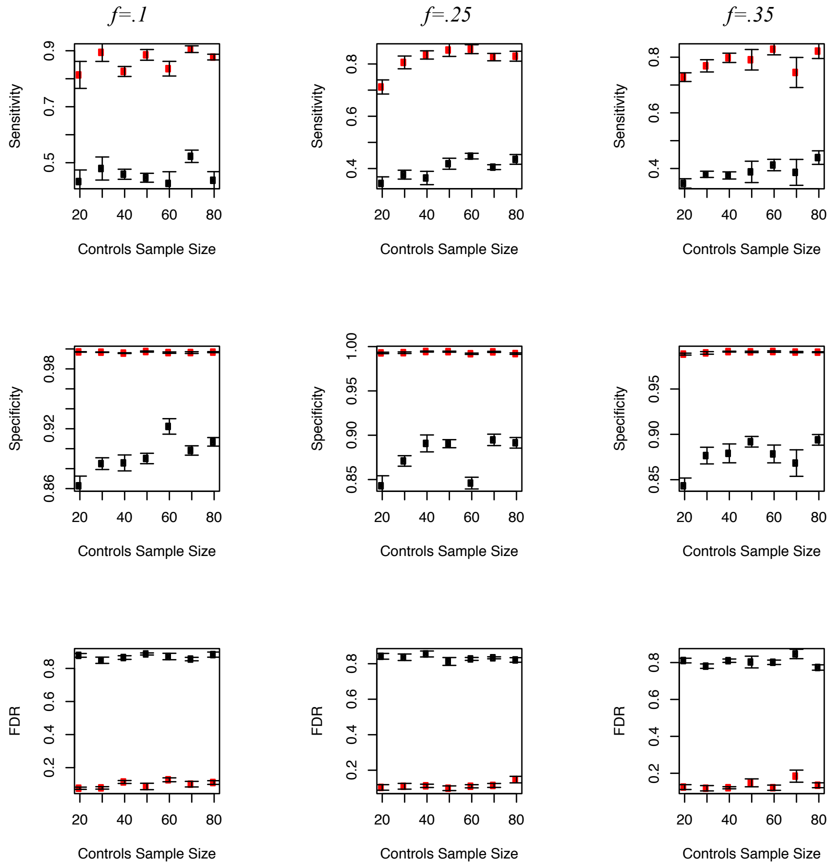

Figure 22: Simulation performance in an unbalanced design. We plot the performance as a function of sample size and fraction of features f that are perturbed in cases. The total number of case samples were fixed to 20, and the number of control samples were varied to simulate unbalanced designs. So in the plot, a sample size of 20 corresponds to a sample size of 20 for the case sample, and therefore reflects a balanced design. The rest represent unbalanced designs. Sample depth fixed to 10K reads on average per sample. Legend: Red, Wrench; Black: TMM.

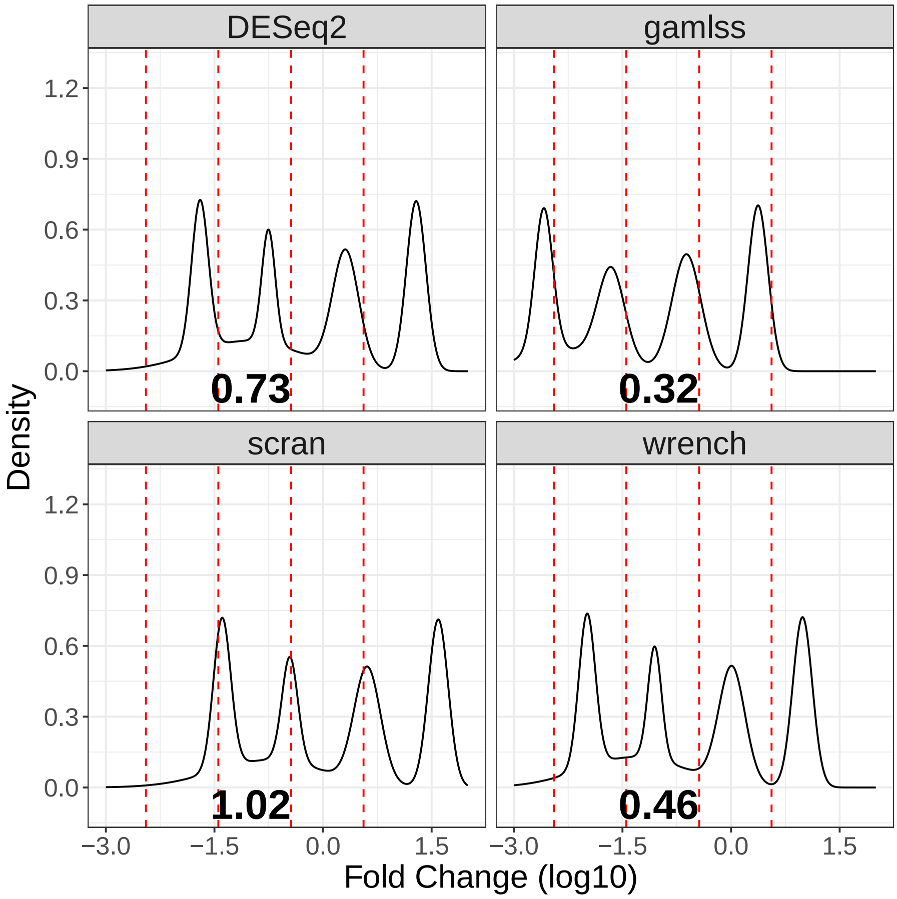

Figure 23: Benchmarking analysis of the Argyropolous et al., miRNA dataset for deviatioin from expected fold changes in the clustered symmetric DE without global changes in expression ratiometric A versus B. Same as Fig. 7 in [14]. The shown numbers measure deviation of the reconstructed fold changes from the true expected fold changes by experimental design, for the pipeline. Lower is better. Refer [14], Fig. 7 for details on experimental design.

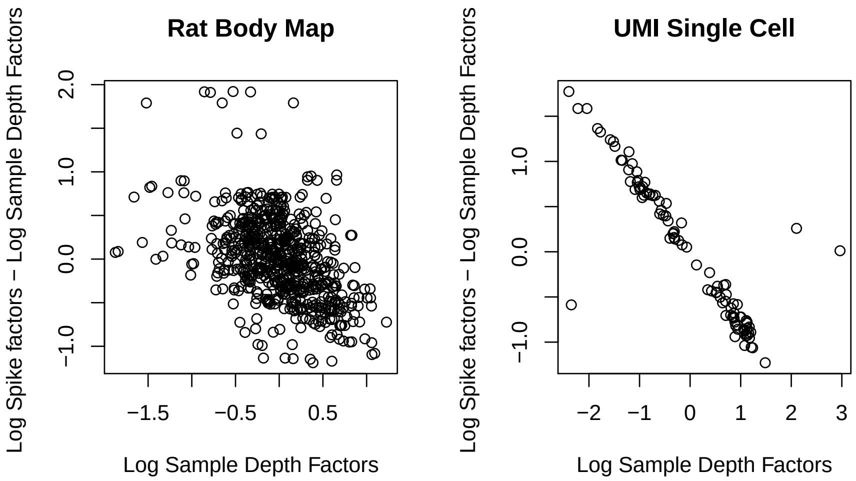

Figure 24: Spike-in proportions show trends with sample depth. The y-axis plots the differences between the logged spike-in count and sample depth, and the x-axis represents sample-depth factors (upto arbitrary scaling).
